# Decapod iridescent virus 1 (DIV1) enters hematopoietic *Cherax quadricarinatus* cells via caveola-mediated endocytosis in a pH-dependent manner

**DOI:** 10.1128/jvi.01681-25

**Published:** 2026-03-30

**Authors:** Qin Zheng, Xiaojuan Chen, Furong Zhao, Junhao Zhang, Jianming Chen

**Affiliations:** 1 Fujian Key Laboratory on Conservation and Sustainable Utilization of Marine Biodiversity, Fuzhou Institute of Oceanography, Minjiang University 26465, Fuzhou, China; Wageningen University & Research, Wageningen, the Netherlands

**Keywords:** decapod iridescent virus 1, *Cherax quadricarinatus*, caveola-mediated endocytosis, internalization mechanism

## Abstract

Decapod iridescent virus 1 (DIV1), the first isolated species of the genus *Decapodiridovirus* (family *Iridoviridae*), is a major pathogen threatening farmed decapods, causing large-scale disease outbreaks and high mortality. However, the mechanisms underlying its cellular entry remain poorly understood. In this study, we investigated DIV1 internalization using hematopoietic tissue cells of *Cherax quadricarinatus*. Pharmacological inhibition assays showed that (i) clathrin-dependent endocytosis inhibitors (pitstop 2, chlorpromazine) failed to suppress DIV1 infection, excluding clathrin-mediated entry; (ii) cellular cholesterol disrupted with methyl-β-cyclodextrin or nystatin significantly inhibited infection, and this effect was reversed by cholesterol replenishment; and (iii) caveolar inhibitors (phorbol 12-myristate 13-acetate, genistein, wortmannin) and organelle pH neutralizers (NH_4_Cl, bafilomycin A1) effectively blocked DIV1 invasion. Fluorescence co-localization and pull-down assays confirmed that DIV1 associates with caveolin-1 during internalization. Additionally, DIV1 entry relies on dynamin and the microtubule cytoskeleton, and post-internalization trafficking of the virus targeted the Golgi apparatus. Collectively, DIV1 enters hematopoietic *C. quadricarinatus* cells via caveola-mediated endocytosis, a process dependent on cholesterol, dynamin, and microtubule cytoskeleton, with subsequent trafficking to the Golgi apparatus.

**IMPORTANCE:**

Elucidating viral entry mechanisms into target cells is fundamental for developing effective antiviral strategies. This study reveals that DIV1, a major threat to decapod aquaculture, utilizes caveola-mediated endocytosis to invade hematopoietic tissue cells of *Cherax quadricarinatus*. This entry process is dependent on membrane cholesterol, dynamin activity, microtubule-based trafficking, and a pH-responsive Golgi environment. These findings fill a critical knowledge gap in *Betairidovirinae* entry biology, distinguish DIV1 from other iridovirids and decapod pathogens (e.g., white spot syndrome virus), and identify key host-derived targets. This work thereby enables the development of targeted antiviral interventions to safeguard decapod aquaculture.

## INTRODUCTION

Clathrin-mediated endocytosis (CME) and caveola-mediated endocytosis (CvME) are two well-studied pathways exploited by numerous viruses for cell entry. In CME, clathrin assembles on the plasma membrane to form clathrin-coated pits, which internalize receptor-bound viruses and bud off as clathrin-coated vesicles. These vesicles are subsequently trafficked to acidic endosomal and lysosomal compartments. By contrast, CvME relies on caveolae, flask-shaped membrane invaginations associated with lipid rafts, that deliver viruses to pH-neutral caveosomes after budding, followed by transport to the Golgi complex or endoplasmic reticulum (1–3). Despite advances in characterizing these pathways, key questions remain regarding virus-specific pathway selection and the translational potential of these pathways as therapeutic targets. Pharmacological inhibition of endocytic processes has emerged as a robust tool to address these questions. It not only dissects virus-host interaction mechanisms (4, 5) but also reveals how pathway selection is regulated, illuminating the intricate crosstalk between viruses and host cells.

Iridovirids, which infect invertebrates and poikilothermic vertebrates, have garnered increasing attention due to their high pathogenicity in aquacultural organisms. The family *Iridoviridae* is classified into two subfamilies: *Alphairidovirinae* (encompassing *Ranavirus*, *Lymphocystivirus*, and *Megalocytivirus*) and *Betairidovirinae* (including *Iridovirus*, *Chloriridovirus*, *Daphniairidovirus*, and *Decapodiridovirus*) (6). Infections by viruses of the subfamily *Alphairidovirinae* often cause host organ failure and mass mortality, leading to substantial aquaculture economic losses. Elucidating iridovirus cellular entry mechanisms is therefore crucial for understanding their pathogenicity and developing novel prevention and control strategies. However, a comprehensive understanding of these mechanisms across the family is lacking, even though entry pathways have been identified for some members. For instance, two *Ranavirus* species, Singapore grouper iridovirus (SGIV) and soft-shelled turtle iridovirus (STIV), enter host cells via CME and macropinocytosis (7–10). In contrast, lymphocystis disease virus (LCDV, *Lymphocystivirus*), infectious spleen and kidney necrosis virus (ISKNV, *Megalocytivirus*), and tiger frog virus (TFV, *Ranavirus*) rely on CvME (11–13). Notably, the cellular entry mechanisms of *Betairidovirinae* viruses remain uncharacterized.

Decapod iridescent virus 1 (DIV1), the only recognized species of the *Decapodiridovirus* genus, induces high mortality in infected decapods (e.g., *Litopenaeus vannamei*, *C. quadricarinatus*, *Exopalaemon carinicauda,* and *Macrobrachium rosenbergii*) (14–19), posing a severe threat to the decapod farming industry. The life cycle of DIV1 has been preliminarily characterized in prior studies (18, 19). Specifically, following host cell infection, DIV1 initiates virion formation within specialized cytoplasmic structures known as virogenic stromata that support viral assembly by housing immature capsids and viral replication machinery. Once assembled, virions then undergo budding from the plasma membrane to acquire an outer envelope. Despite these insights into downstream stages of the viral life cycle, a critical upstream step, the specific mechanism by which DIV1 initially enters host cells to establish infection, remains largely uncharacterized, representing a key gap in our understanding of this pathogenic virus.

To address the knowledge gap, we focused on CME and CvME, the two most common entry pathways for other iridovirids, and systematically analyzed the entry of DIV1 into hematopoietic tissue (HPT) cells from *C. quadricarinatus* by combining drug inhibition assays, fluorescence co-localization experiments, and pull-down assays. Our results demonstrate that DIV1 enters HPT cells via a CvME pathway, which is strictly dependent on cholesterol and dynamin activity. These findings not only clarify the initial stages of DIV1 infection but also provide a theoretical basis for future antiviral research, potentially facilitating the development of targeted therapies against DIV1 and related *Betairidovirinae* viruses.

## RESULTS

### Dynamics of DIV1 entry into *C. quadricarinatus* HPT cells

**Fig 1:**
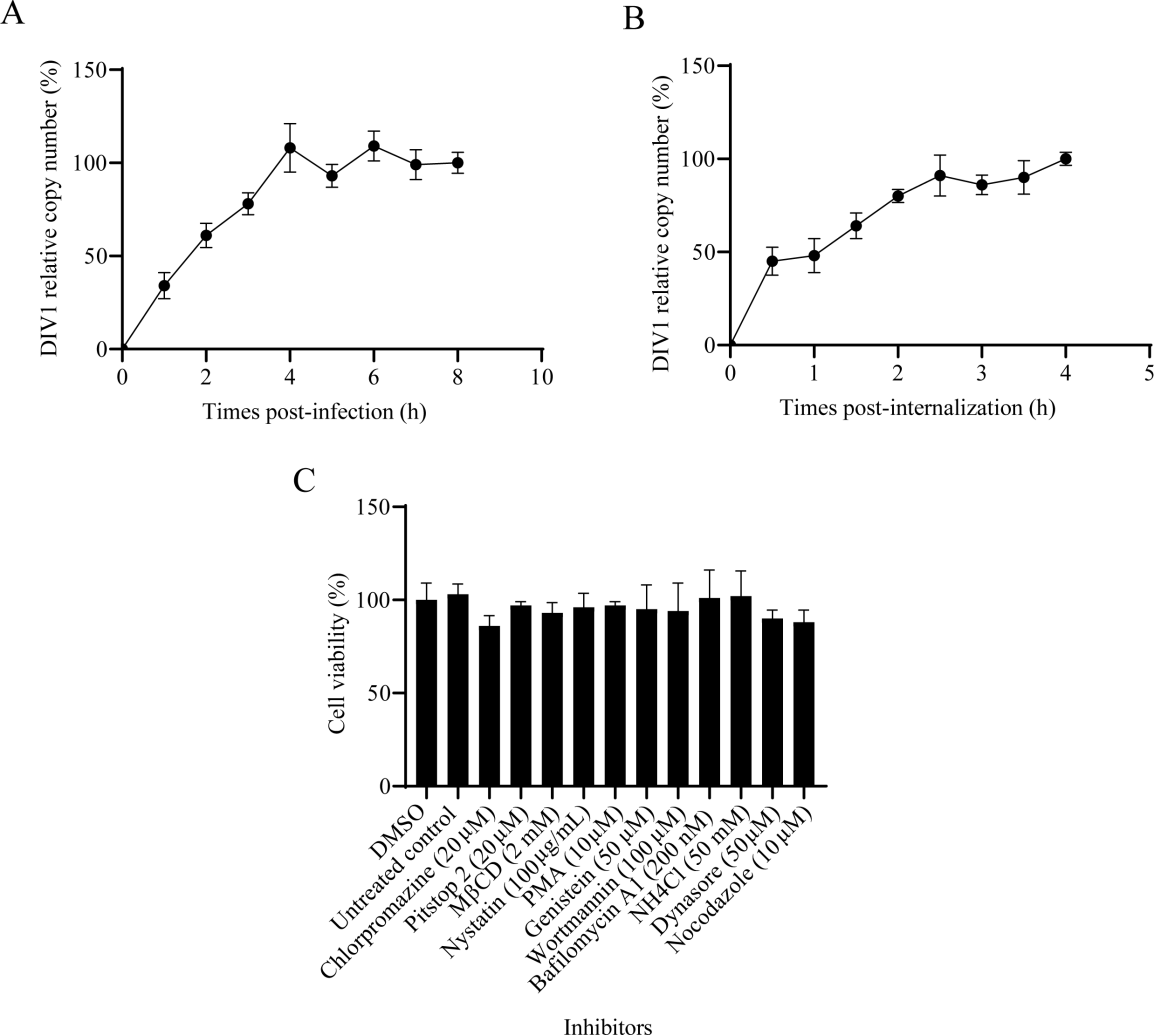
Dynamics of DIV1 entry into *C. quadricarinatus* HPT cells and evaluation of cell viability following the inhibitor treatment. (**A**) DIV1 infection kinetics in HPT cells. Cells were infected with DIV1. At preset time points post-infection, non-internalized viruses were inactivated using citrate buffer. Infected cells were then cultured continuously until 72 hpi. (**B**) DIV1 entry kinetics into HPT cells. Cells were incubated with DIV1 at 4°C for 1 h (to allow virus binding to the cell surface without initiating endocytosis). Unbound viruses were removed by washing with pre-cooled PBS, and cells were transferred to 27°C to trigger viral entry. At the indicated time point after shifting to 27°C, non-internalized viruses were inactivated with citrate buffer, and cells were cultured until 72 hpi. For panels (**A**) and (**B**), viral copy numbers in the culture medium were quantified by qPCR. The copy number at 8 hpi (**A**) or 4 h post-initiation of internalization (**B**) was normalized to 100%, and the relative percentages of viral copies at other time points were calculated accordingly. Data are presented as the mean ± standard deviations (SD) from three independent experiments. (**C**) Viability of HPT cells treated with inhibitors at their MNTCs. To determine the MNTCs of each inhibitor in HPT cells, we first performed a pre-experimental screening (testing five concentration gradients per inhibitor; data not shown) to exclude toxic doses. Subsequently, cells were incubated with pre-determined MNTCs for 5 h at 27°C: chlorpromazine (20 μm), pitstop 2 (20 μm), MβCD (2 mM), nystatin (100 μg/mL), PMA (10 μM), genistein (50 μM), wortmannin (100 μM), bafilomycin A1 (200 nM), NH_4_Cl (50 mM), dynasore (50 μM), and nocodazole (10 μM). Control cells included both solvent-treated groups (receiving an equal volume of DMSO or ddH2O) and an untreated blank control group. After inhibitor treatment, the medium was replaced with fresh medium containing 10 μL CCK-8 reagent per well, and cells were incubated for an additional 2 h. A blank control group (containing only fresh medium and 10 μL CCK-8, without cells) was set up to correct for background absorbance. Cell viability was determined by measuring OD_450_. Data are representative of three independent experiments and presented as the means ± SD.

Our previous study has confirmed that HPT cells from *C. quadricarinatus* are susceptible to DIV1 infection (20). To elucidate the cellular entry mechanism of DIV1, we first characterized the dynamics of viral infection and entry in HPT cells. To analyze the kinetics of DIV1 infection, HPT cells were inoculated with DIV1. At 0, 1, 2, 3, 4, 5, 6, 7, and 8 hours post-infection (hpi), non-internalized virions were removed from the cell surface. Cells were then continuously incubated until 72 hpi, after which the copy number of DIV1 in the culture medium was quantified via quantitative polymerase chain reaction (qPCR). For normalization, the viral copy number at 8 hpi was set at 100%, and the relative percentages of viral copies at other timepoints were calculated accordingly. As shown in Fig. 1A, the relative copy number of DIV1 exhibited an overall upward trend with the extension of post-infection time. It reached a relatively high level at approximately 4 hpi and remained within this high range thereafter, despite minor fluctuations.

To investigate DIV1 entry kinetics, cells were pre-chilled and inoculated with DIV1 virions at 4°C for 1 h to allow viral attachment but suppress internalization. Cells were then transferred to 27°C to initiate the internalization of surface-bound virions. At 0, 0.5, 1, 1.5, 2, 2.5, 3, 3.5, and 4 h post-internalization initiation, non-internalized virions were inactivated to ensure only internalized virions were detected. Cells were cultured until 72 hpi, and qPCR was employed to quantify DIV1 copy number in the culture medium. The viral copy level at 4 h post-internalization was set as 100% for normalization, and relative percentages of other time point groups were calculated correspondingly. As shown in Fig. 1B, the relative copy number of DIV1 increased gradually with the extension of post-internalization time, showing a continuous growth trend. Notably, the relative copy number approached 100% at 2 h post-internalization, indicating that most bound virions had completed internalization by this time.

A critical prerequisite for reliable inhibitor-based entry assays is ensuring that observed effects on viral infection are not confounded by non-specific inhibitor-induced cell damage. We therefore first evaluated the cytotoxicity of all candidate inhibitors using the CCK-8 assay. Based on recommendations in reagent instructions and concentrations commonly used in endocytosis-related studies (7, 11–13, 21), we first conducted preliminary experiments to screen concentration gradients for each inhibitor, aiming to determine their maximum non-toxic concentrations (MNTCs) in HPT cells. For the pre-experimental screening, a gradient of five concentrations was tested for each inhibitor (data not shown). We finally determined the MNTCs of each inhibitor in HPT cells as follows: chlorpromazine (20 μM), pitstop 2 (20 μM), MβCD (2 mM), nystatin (100 μg/mL), PMA (10 μM), genistein (50 μM), wortmannin (100 μM), bafilomycin A1 (200 nM), NH_4_Cl (50 mM), dynasore (50 μM), and nocodazole (10 μM). At these concentrations, cell viability was comparable to that of the solvent-treated group, with no statistically significant differences (Fig. 1C), confirming that subsequent inhibitor treatments would not interfere with experimental results via cell toxicity. In addition, there was no statistically significant variation in cell viability between the solvent-treated group and the untreated control group, indicating that the solvent had no influence on cell viability.

### DIV1 entry and infection are clathrin-independent

CME is a well-characterized entry pathway for many viruses, such as Junin arenavirus and Hepatitis C virus (4, 22). To determine whether DIV1 utilizes CME for cellular entry, we treated HPT cells with two CME-specific inhibitors, pitstop 2 and chlorpromazine, and assessed their effects on DIV1 entry.

**Fig 2:**
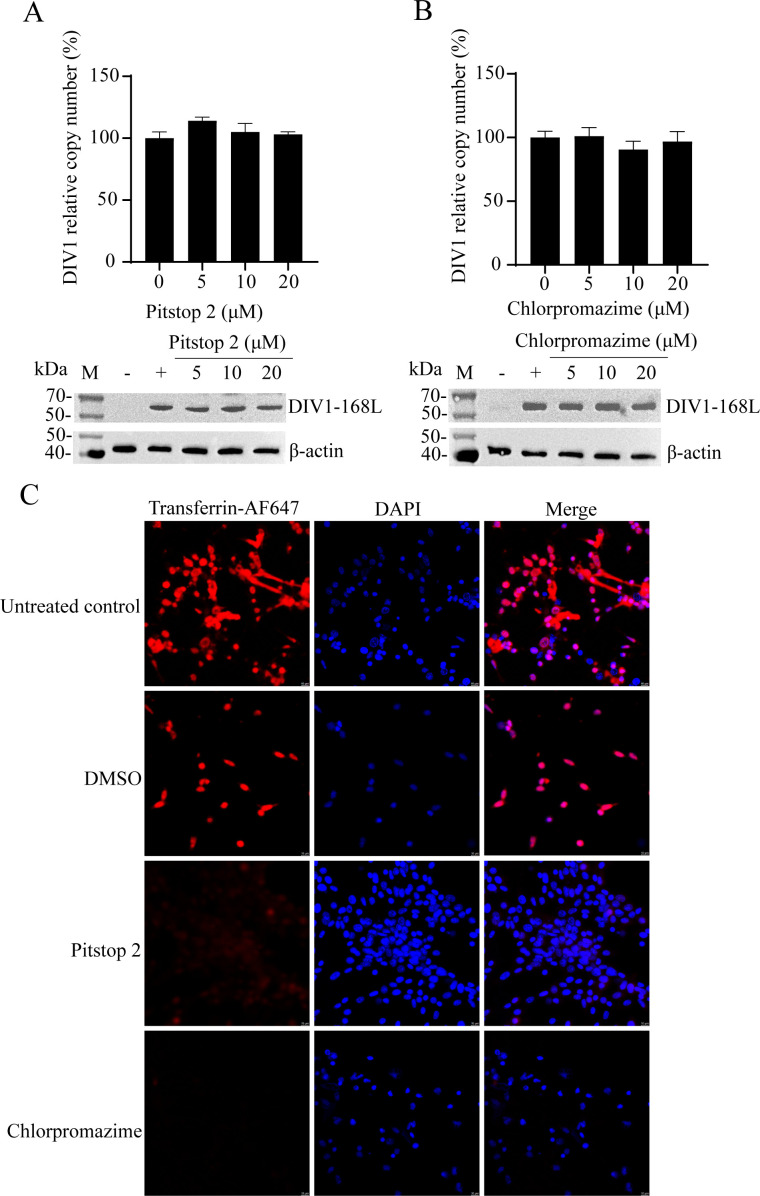
DIV1 infection is independent of CME. (**A and B**) Effects of CME inhibitors on DIV1 infection. HPT cells were pretreated with gradient concentrations of the CME inhibitors pitstop 2 (**A**) and chlorpromazine (**B**) for 1 h. After pretreatment, DIV1 was added to the cells for 4 h of incubation, followed by 72 h of continued culture. Two control groups were included: DIV1-infected cells without inhibitor pretreatment (positive control, “+”) and non-DIV1-infected cells (negative control, “−”). The cell culture system was centrifuged, and DIV1 infection efficiency was evaluated via two approaches: WB using a DIV1-168L-specific antibody (to detect DIV1-168L expression in the cell pellet) and qPCR (to quantify DIV1 copy numbers in the supernatant). The viral copy number of the untreated positive control group was normalized to 100%, and the relative percentages of viral copies in inhibitor-treated groups were calculated accordingly. Endogenous β-actin served as the loading control. Data are presented as the means ± SD from three independent experiments. (**C**) Validation of CME inhibition by pitstop 2 and chlorpromazine. HPT cells were pretreated with 20 μM pitstop 2 or 20 μM chlorpromazine at 27°C for 1 h, then incubated with transferrin-AF647 for an additional 1 h at 27°C. Cells treated with DMSO or left untreated, both incubated with transferrin-AF647, served as the controls to assess normal CME activity. The internalization of transferrin-AF647 was detected by fluorescence microscopy.

Pitstop 2 blocks clathrin-coated pit formation by disrupting interactions between the clathrin heavy chain and adaptor proteins (23). Cells were treated with 5, 10, or 20 μM pitstop 2 prior to DIV1 infection. At 72 hpi, the cell culture system was centrifuged to separate the supernatant (culture medium) and cell pellet. Viral copy numbers in the culture medium were quantified by qPCR to assess released virions, while the cell pellet was processed for WB to detect the expression of DIV1-168L using a validated specific antibody (24). As a core envelope protein of DIV1, DIV1-168L is encoded by the late gene *168L* of DIV1 and predicted to be a myristoylated membrane protein (24), and its expression level is closely associated with viral entry efficiency. As shown in Fig. 2A, statistical analysis revealed no significant differences in either viral copy numbers or DIV1-168L protein expression between pitstop 2-treated groups and the control, indicating CME inhibition via pitstop 2 does not impair DIV1 entry.

To validate this finding, we performed complementary experiments with chlorpromazine, another CME inhibitor that interferes with clathrin assembly, disrupts its interactions with associated proteins, and alters cell membrane fluidity (25). Consistent with the pitstop 2 results, qPCR and WB analyses showed chlorpromazine had no effect on DIV1 entry, even at 20 μM (Fig. 2B).

Transferrin is exclusively internalized via CME (26), making it an ideal positive control to verify inhibitor efficacy. To confirm pitstop 2 and chlorpromazine effectively inhibited CEM, HPT cells pretreated with these inhibitors were incubated with transferrin-AF647 for 1 h. Transferrin internalization was visualized by confocal microscopy. As shown in Fig. 2C, no significant differences in transferrin internalization were observed between the DMSO-treated control group and the untreated blank control group, which ruled out the possibility that the solvent itself could interfere with endocytosis. Additionally, both inhibitors significantly reduced transferrin-AF647 internalization compared with the control, confirming successful CME suppression in our experimental system. Collectively, these results demonstrate that DIV1 entry is clathrin-independent.

### Cholesterol-dependent entry of DIV1 into HPT cells

Since CME was ruled out as DIV1’s entry route, we next investigated the potential involvement of CvME, a major endocytic pathway dependent on lipid rafts. Caveolae, the core structural components of CvME, are highly enriched in cholesterol. Eliminating or disrupting cholesterol directly impaired or abolished CvME. To determine whether DIV1 utilizes CvME for entry, we first disrupted membrane cholesterol homeostasis using two distinct reagents (MβCD and nystatin), then validated their effects on viral entry and pathway specificity.

**Fig 3:**
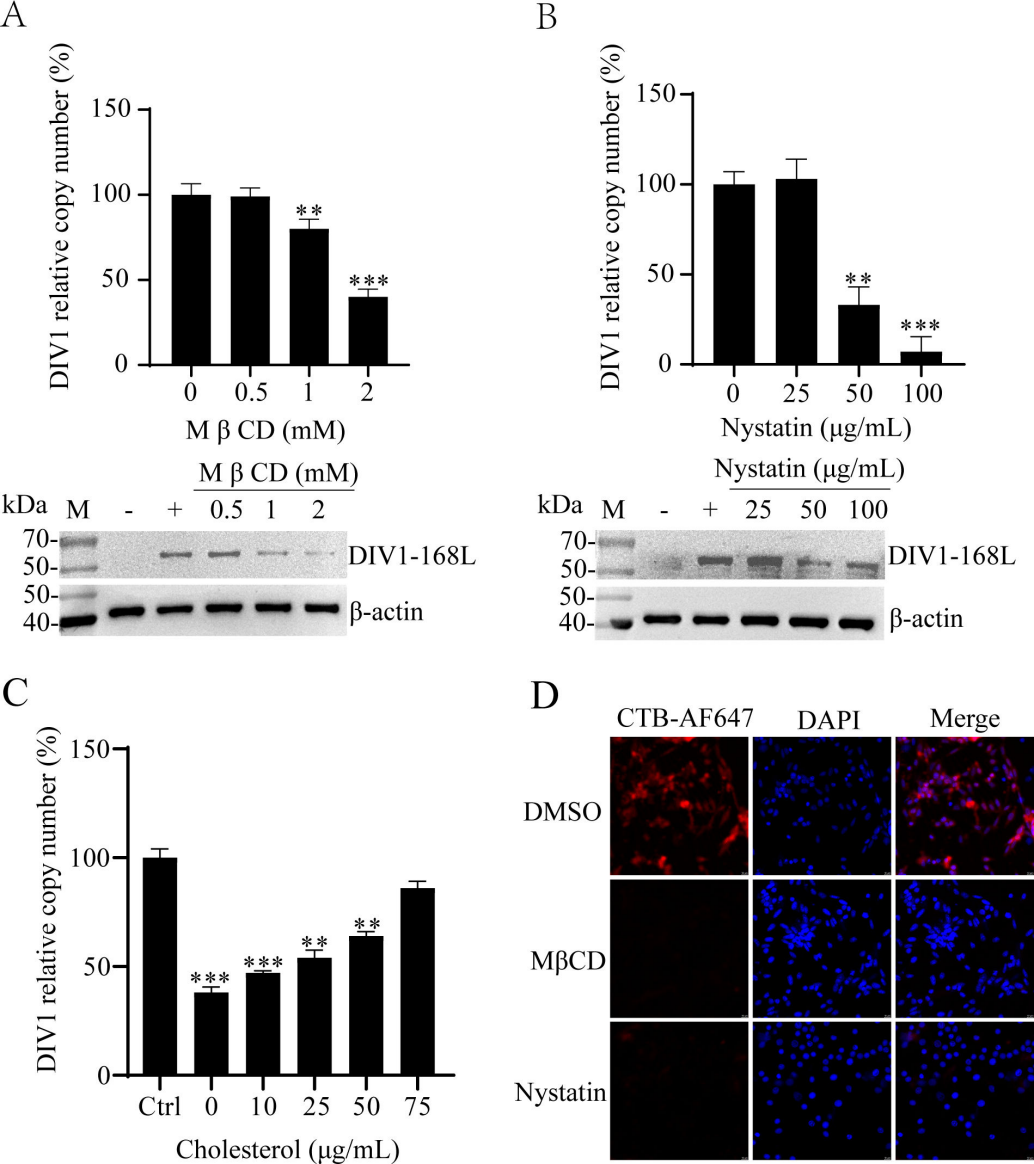
DIV1 infection depends on membrane cholesterol. (**A and B**) Effects of cholesterol inhibitors on DIV1 infection. HPT cells were pretreated with gradient concentrations of MβCD (**A**) or nystatin (**B**) for 1 h at 27°C. After pretreatment, DIV1 was added to the cells for 4 h of incubation, followed by 72 h of continued culture. DIV1-infected cells without inhibitor pretreatment served as the positive control (“+”), and non-DIV1-infected cells as the negative control (“−”). DIV1 infection efficiency was evaluated via WB using a DIV1-168L-specific antibody and qPCR to determine DIV1 copy numbers in the culture medium. The viral copy number of the untreated positive control group was normalized to 100%, and the relative percentages of viral copies in inhibitor-treated groups were calculated accordingly. Endogenous β-actin served as the loading control. (**C**) Cholesterol replenishment assay in DIV1-infected cells pretreated with MβCD. Cells were pretreated with 2 mM MβCD, followed by the addition of various concentrations of exogenous cholesterol before DIV1 infection. Subsequently, cells were subjected to the same DIV1 infection and culture protocol as described in (**A and B**). Viral copy numbers in the culture medium were quantified via qPCR. For normalization, the viral copy number of the positive control (DIV1-infected without any reagent treatment) was set to 100% as the reference for relative quantification. Data are presented as the mean ± SD from three independent experiments. Statistical significance was analyzed using Student’s t-test, with *P < 0.05, **P < 0.01, and ***P < 0.001 indicating significant differences compared to the positive control group. (**D**) Validation of caveola-mediated endocytosis inhibition by MβCD and nystatin. HPT cells were pretreated with MβCD (2 mM) or nystatin (100 μg/mL) at 27°C for 1 h, then incubated with CTB-AF647 for an additional 1 h at 27°C. Cells treated with the solvent (DMSO) and incubated with CTB-AF647 served as the control. The internalization of CTB-AF647 was detected via fluorescence microscopy.

MβCD depletes membrane cholesterol by extracting it from the lipid bilayer, thereby preventing caveola formation and abrogating CvME activity (27). To assess its effect on DIV1 entry, HPT cells were pretreated with MβCD prior to DIV1 inoculation. At 72 hpi, qPCR quantified viral particle abundance in culture medium, and WB detected intracellular levels of the DIV1-168L. As shown in Fig. 3A, MβCD inhibited DIV1 infection dose-dependently. Compared with the untreated control, 1 mM MβCD reduced viral particle counts to ~57%, while 2 mM MβCD further reduced them to ~29%. Consistent with qPCR results, WB analysis revealed a progressive reduction in intracellular DIV1-168L levels with increasing MβCD concentrations, confirming that cholesterol depletion impairs DIV1 entry and subsequent replication.

To further validate the role of CvME in DIV1 entry, we used nystatin, a polyene antibiotic that binds membrane cholesterol, disrupts lipid raft structure (without depleting cholesterol) (28), and specifically interferes with CvME. HPT cells were pretreated with nystatin before DIV1 infection. At 72 hpi, viral load and DIV1-168L levels were analyzed. qPCR results (Fig. 3B) showed that nystatin also inhibited DIV1 infection in a dose-dependent manner: 50 μM nystatin reduced viral particle counts to ~36% of the control, while 100 μM nystatin reduced them to ~16%. WB analysis corroborated these findings: intracellular DIV1-168L accumulation gradually diminished with increasing nystatin concentrations, consistent with reduced viral particle. These data further support that DIV1 entry depends on cholesterol and CvME.

To confirm that MβCD’s inhibitory effect was specifically due to cholesterol depletion, we performed cholesterol replenishment experiments. HPT cells were co-incubated with 2 mM MβCD and gradient concentrations of exogenous cholesterol prior to DIV1 inoculation. Viral particle counts were analyzed at 72 hpi. Quantitative analysis showed that DIV1 infection rate increased significantly from ~29% to ~83% after cholesterol supplementation, with the recovery of infection rate enhanced in a dose-dependent manner as the cholesterol concentration increased (Fig. 3C). This confirms MβCD’s inhibition of DIV1 infection is closely linked to cholesterol depletion, and cholesterol replenishment can effectively reverse this effect.

To confirm MβCD and nystatin effectively perturbed cellular cholesterol homeostasis, we used cholera toxin subunit B (CTB) internalization, a well-established positive control for CvME function. CTB specifically binds to ganglioside GM1 (a lipid raft component), is internalized exclusively via CvME, and disruption of cholesterol homeostasis impairs this internalization (29). Fluorescence analysis showed that, relative to the untreated control, cells treated with MβCD or nystatin exhibited significantly reduced intracellular red fluorescence intensity (Fig. 3D), indicating impaired CTB internalization. This confirms that both reagents effectively disrupted cholesterol-dependent lipid raft structure and CvME activity under our experimental conditions.

Collectively, these results demonstrate that depleting or sequestering of membrane cholesterol inhibits DIV1 entry into HPT cells, cholesterol replenishment reverses MβCD-mediated inhibition, and disrupting lipid raft structure abrogates DIV1 entry, which strongly indicates that DIV1 enters HPT cells via CvME and that cholesterol-rich lipid rafts (particularly caveolae) are critical for this process.

### DIV1 entry into HPT cells is caveola-dependent

After DIV1 is enveloped by caveolae at the cell membrane, caveolae detach from the membrane through the coordinated action of multiple proteins and signaling molecules. This detachment process is closely linked to the phosphorylation of caveolin and adaptor proteins and is regulated by key signaling components, including phosphatidylinositol 3-kinase (PI3K) and mitogen-activated protein kinase family (30). Notably, inhibiting this phosphorylation cascade effectively blocks caveola internalization, thereby disrupting CvME. To clarify the role of CvME in DIV1 entry into HPT cells, we evaluated the effects of three CvME-specific inhibitors on caveola internalization.

**Fig 4:**
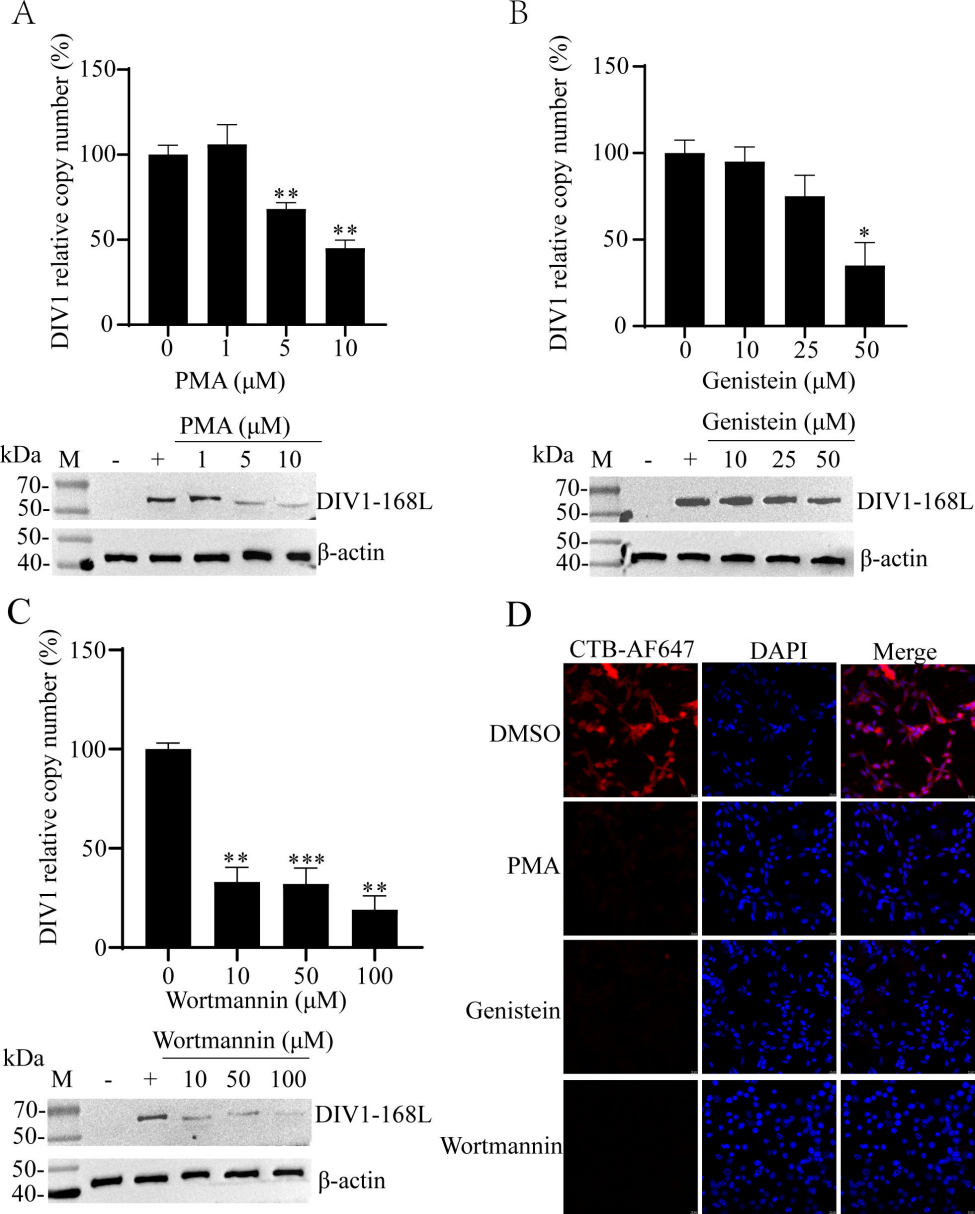
DIV1 infection depends on CvME. (**A–C**) Effects of CvME-modulating reagents on DIV1 infection. HPT cells were pretreated with gradient concentrations of PMA (**A**), genistein (**B**), and wortmannin (**C**) for 1 h. After pretreatment, DIV1 was added to the cells for 4 h of incubation, followed by 72 h of continued culture. DIV1-infected cells without inhibitor pretreatment served as the positive control (“+”), and non-DIV1-infected cells as the negative control (“−”). DIV1 infection efficiency was evaluated via WB using a DIV1-168L-specific antibody and qPCR to determine DIV1 copy numbers in the culture medium. The viral copy number of the untreated positive control group was normalized to 100%, and the relative percentages of viral copies in inhibitor-treated groups were calculated accordingly. Endogenous β-actin served as the loading control. Data are presented as the mean ± SD from three independent experiments. Statistical significance was analyzed using Student’s t-test, with *P < 0.05, **P < 0.01, and ***P < 0.001 indicating significant differences compared to the positive control group. (**D**) Validation of the effects of PMA, genistein, and wortmannin on CvME. HPT cells were pretreated with PMA (10 μM), genistein (50 μM), or wortmannin (100 μM) at 27°C for 1 h, then incubated with CTB-AF647 for an additional 1 h at 27°C. Cells treated with DMSO and incubated with CTB-AF647 served as the control to reflect normal CvME activity. The internalization of CTB-AF647 was detected via fluorescence microscopy.

First, we used PMA, a well-characterized modulator of CvME. At high concentrations, PMA inhibits this pathway by hyperactivating protein kinase C (PKC), thereby disrupting caveolae structure and function (31). As shown in Fig. 4A, PMA treatment caused a dose-dependent reduction in DIV1 infectivity. At concentrations of 5 μM and 10 μM, DIV1 copy numbers were significantly lower than those in the 0 μM (control) group. WB analysis further confirmed this trend, showing that the levels of the viral protein DIV1-168L decreased with increasing PMA concentrations, while the internal control (β-actin) remained stably expressed. These results align with PMA’s known role in inhibiting CvME, supporting the involvement of CvME in DIV1 entry.

Next, we tested genistein, a well-documented inhibitor of receptor tyrosine kinases (RTKs). Genistein impairs CvME efficiency by abrogating RTK-mediated downstream signaling cascades; specifically, RTK inhibition blocks the activation of Src family kinases and subsequent phosphorylation of caveolin-1, a key step required for caveolae formation and endocytic initiation (30). Over the concentration range of 0–50 μM, genistein induced a dose-dependent decrease in DIV1 infectivity. At 50 μM, the DIV1 copy number was reduced to ~31% of the control. WB analysis showed a gradual decline in DIV1-168L levels with increasing genistein concentrations, with a notable reduction in the amount of DIV1-168L detected at 50 μM, while β-actin expression remained unchanged across all groups (Fig. 4B). This confirms that RTK signaling, a key regulatory pathway for CvME, is required for DIV1 entry.

We then assessed wortmannin, a potent and specific PI3K inhibitor that suppresses CvME by targeting PI3K enzymatic activity (32, 33). Treatment with wortmannin (10 μM, 50 μM, and 100 μM) resulted in a dose-dependent reduction in DIV1 infectivity, accompanied by a corresponding decrease in DIV1-168L levels (Fig. 4C). The stable expression of β-actin ruled out non-specific effects of wortmannin, indicating that PI3K-dependent CvME contributes to DIV1 entry.

To validate that these inhibitors specifically target CvME, we further evaluated their effects on the internalization of CTB. Compared with the control group, cells treated with PMA, genistein, or wortmannin showed significantly reduced cellular fluorescence intensity (Fig. 4D), confirming that these compounds effectively inhibit CvME.

Collectively, these data demonstrate that CvME is a key pathway for DIV1 entry into hematopoietic *C. quadricarinatus* cells, and its activity is regulated by multiple signaling mechanisms (PKC, RTKs, and PI3K). Inhibition of these signaling pathways suppresses DIV1 infection, providing critical insights for the development of antiviral strategies.

### pH dependence of DIV1 entry into HPT cells

Canonical CvME is characterized as a pH-independent process; however, CvME involving the trans-Golgi network exhibits distinct pH dependence, a feature exploited by multiple viruses (e.g., BK virus, TFV) for cellular entry (11, 34). To investigate whether DIV1 entry into HPT cells relies on pH, we used two pharmacological inhibitors, bafilomycin A1 and NH_4_Cl, to block the acidification of intracellular organelles (5). The impact of organelle acidification on DIV1 infection was then evaluated using qPCR and WB.

**Fig 5:**
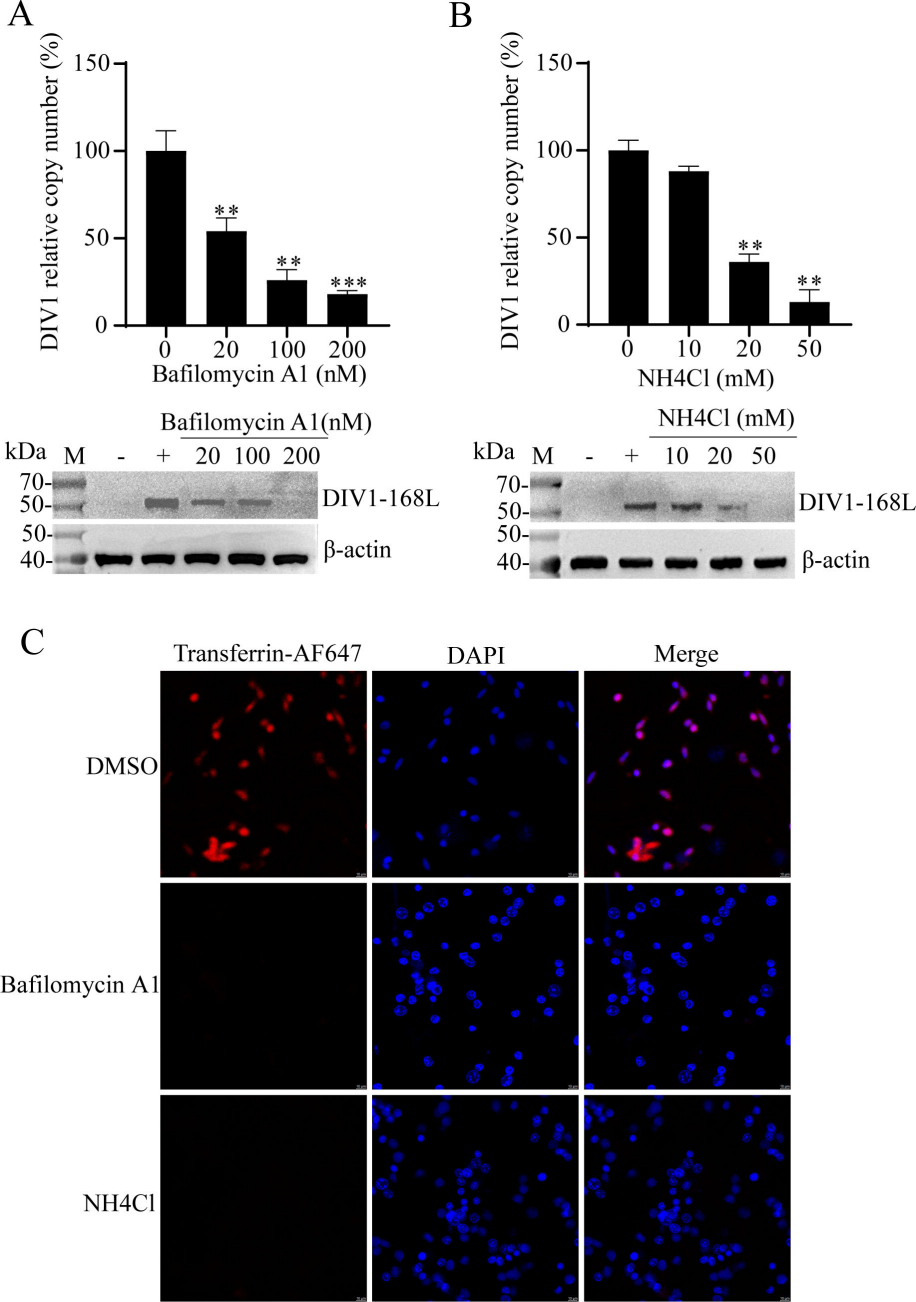
DIV1 infection depends on organelle acidification. (**A and B**) Effects of organelle acidification inhibitors on DIV1 infection. HPT cells were pretreated with gradient concentrations of bafilomycin A1 (**A**) and NH_4_Cl (**B**) for 1 h. After pretreatment, DIV1 was added to the cells for 4 h of incubation, followed by 72 h of continued culture. Two control groups were included: DIV1-infected cells without inhibitor pretreatment (positive control, “+”) and non-DIV1-infected cells (negative control, “−”). DIV1 infection efficiency was evaluated via two approaches: WB using a DIV1-168L-specific antibody and qPCR (to quantify DIV1 copy numbers in the culture medium). The viral copy number of the untreated positive control groups was normalized to 100%, and the relative percentages of viral copies in inhibitor-treated groups were calculated accordingly. Endogenous β-actin served as the loading control. Data are presented as the mean ± SD from three independent experiments. Statistical significance was analyzed using Student’s t-test, with **P < 0.01 and ***P < 0.001 indicating significant differences compared to the positive control group. (**C**) Validation of the effects of bafilomycin A1 and NH_4_Cl on organelle acidification. HPT cells were pretreated with bafilomycin A1 (200 nM) or NH_4_Cl (50 mM) at 27°C for 1 h, then incubated with transferrin-AF647 for an additional 1 h at 27°C. Cells treated with DMSO and incubated with transferrin-AF647 served as the control to reflect normal pH-dependent endocytosis.

Treatment with either bafilomycin A1 or NH_4_Cl effectively inhibited the acidification of intracellular organelles. For bafilomycin A1, a concentration-dependent reduction in the relative copy number of DIV1 was observed (Fig. 5A). Compared to the untreated control (0 nM), viral loads decreased to 57%, 26%, and 19% at concentrations of 20 nM, 100 nM, and 200 nM, respectively. Consistently, NH_4_Cl treatment also induced a concentration-dependent decline in the DIV1 relative copy number, with 20 mM and 50 mM NH_4_Cl reducing viral loads to 18% and 9% of the untreated control (0 mM), respectively (Fig. 5B). WB analysis further confirmed that the expression level of the DIV1-168L protein was markedly lower in both NH_4_Cl^−^ and bafilomycin A1-treated groups than in the control.

To validate that the inhibitors effectively disrupted intracellular organelle acidification (a key step in pH-dependent endocytic processes), we assessed transferrin internalization (Fig. 5C). Relative to the negative control, treatment with bafilomycin A1 or NH_4_Cl significantly reduced transferrin internalization in HPT cells, confirming the efficacy of the inhibitor in blocking organelle acidification.

Collectively, these results demonstrate that inhibiting intracellular organelle acidification impairs the efficiency of DIV1 entry into HPT cells, confirming that DIV1 entry into HPT cells is pH-dependent.

### Dynamin and microtubule dependence of DIV1 entry

**Fig 6:**
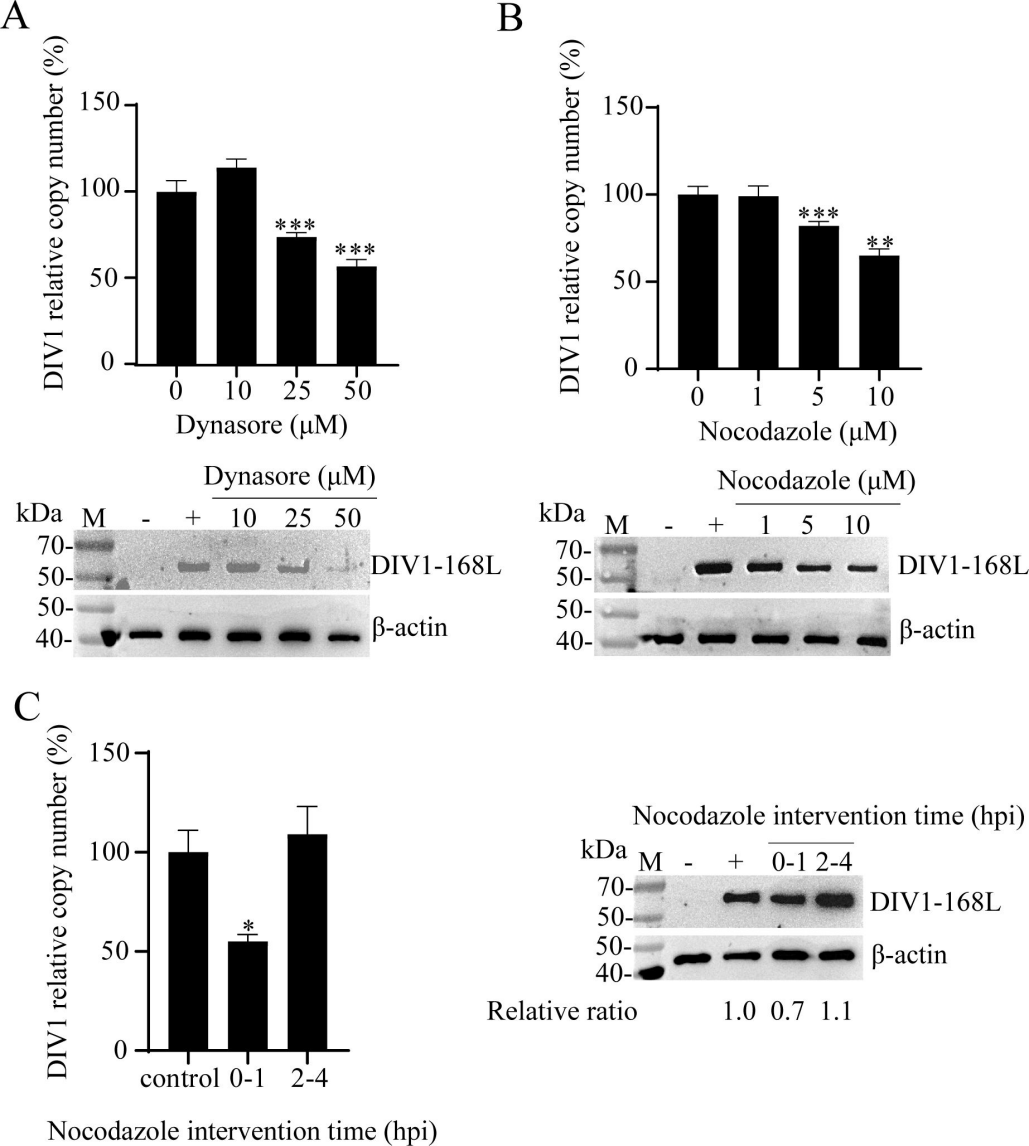
DIV1 infection relies on dynamin and microtubule-dependent processes. (**A and B**) Effects of dynamin inhibitor dynasore and microtubule inhibitor nocodazole on DIV1 infection. HPT cells were pretreated with gradient concentrations of dynasore (**A**) and nocodazole (**B**) for 1 h. After pretreatment, DIV1 was added for 4 h of incubation, followed by 72 h of continued culture. Two control groups were included: DIV1-infected cells without inhibitor pretreatment (positive control, “+”) and non-DIV1-infected cells (negative control, “−”). (**C**) Effects of nocodazole intervention time windows on DIV1 infection efficiency. HPT cells were divided into positive control (no nocodazole), intervention group 1 (0–1 hpi: nocodazole (10 μM) added at 0 hpi and removed at 1 hpi), and intervention group 2 (2–4 hpi: nocodazole (10 μM) added at 2 hpi and removed at 4 hpi). Viral infection was evaluated via WB using a DIV1-168L-specific antibody and qPCR to determine DIV1 copy numbers in the culture medium. The copy number of untreated positive control cells was normalized to 100%, and inhibitor-treated groups were calculated as relative percentages. Endogenous β-actin was used as the loading control. Data are presented as the mean ± SD from three independent experiments. Statistical significance was analyzed using Student’s t-test, with *P < 0.05, **P < 0.01, and ***P < 0.001 indicating significant differences compared to the positive control group.

Dynamin is a key motor protein that mediates vesicle scission from the cell surface in both CME and CvME (35). Dynasore, a specific dynamin inhibitor, suppresses the GTPase activity of dynamin-1 and dynamin-2, thereby blocking dynamin-dependent endocytic processes (36). To evaluate whether dynamin is required for DIV1 entry and early infection, HPT cells were pretreated with gradient concentrations of dynasore, and the inhibitor’s effect on DIV1 infection was analyzed via qPCR and WB. As shown in Fig. 6A, relative to the control group (0 μM dynasore), exposure to 10 μM dynasore caused a marginal, non-significant increase in the DIV1 relative copy number. In stark contrast, at concentrations of 25 μM and 50 μM, dynasore dramatically reduced the DIV1 relative copy number, suggesting that supraphysiological concentrations of dynasore inhibit DIV1 replication. WB assays further confirmed this trend. The abundance of the viral protein DIV1-168L decreased in a dose-dependent manner with increasing dynasore concentrations, consistent with the qPCR results. β-actin served as a loading control, and its expression remained stable across all experimental groups. These data demonstrate that 25 μM and 50 μM dynasore effectively inhibit DIV1 infection, confirming a dynamin-dependent mechanism for DIV1 entry.

The classical CvME pathway relies on microtubules, which function as transport tracks for caveolar vesicles (2). To investigate the role of microtubules in DIV1 entry, cells were treated with nocodazole, a microtubule-depolymerizing agent. First, we assessed the DIV1 relative copy number. As shown in Fig. 6B, compared with the control group (0 μM nocodazole), 1 μM nocodazole had no significant effect on viral copy number. However, when the nocodazole concentration was increased to 5 μM and 10 μM, the DIV1 relative copy number was significantly reduced, indicating that higher concentrations of nocodazole inhibit DIV1 replication. WB results corroborated this pattern: the expression level of DIV1-168L decreased in a dose-dependent manner with increasing nocodazole concentrations, matching the qPCR-derived trend.

To further define the critical time window during which microtubules function in DIV1 infection, we performed time-specific intervention experiments with nocodazole. As shown in Fig. 6C, compared with the control group (no nocodazole treatment), the DIV1 relative copy number was significantly reduced when nocodazole was added during the 0–1 hpi period. In contrast, nocodazole intervention during the 2–4 hpi period had no obvious inhibitory effect, with viral DNA copy numbers remaining close to those of the control. Corresponding WB results showed consistent trends: when normalized to the internal reference β-actin (with the control group set to a relative ratio of 1.0), the expression level of the DIV1-168L protein was downregulated in the 0–1 hpi intervention group (relative ratio = 0.7), while it remained stable in the 2–4 hpi group (relative ratio = 1.1).

These results demonstrate that nocodazole-mediated microtubule depolymerization effectively blocks DIV1 infection. Moreover, the early stage of infection (0–1 hpi) is identified as the critical window for microtubule-dependent transport during DIV1 entry, highlighting an essential role for microtubules in this process.

### DIV1 co-localizes with caveolin-1 and the Golgi apparatus but not with transferrin

**Fig 7:**
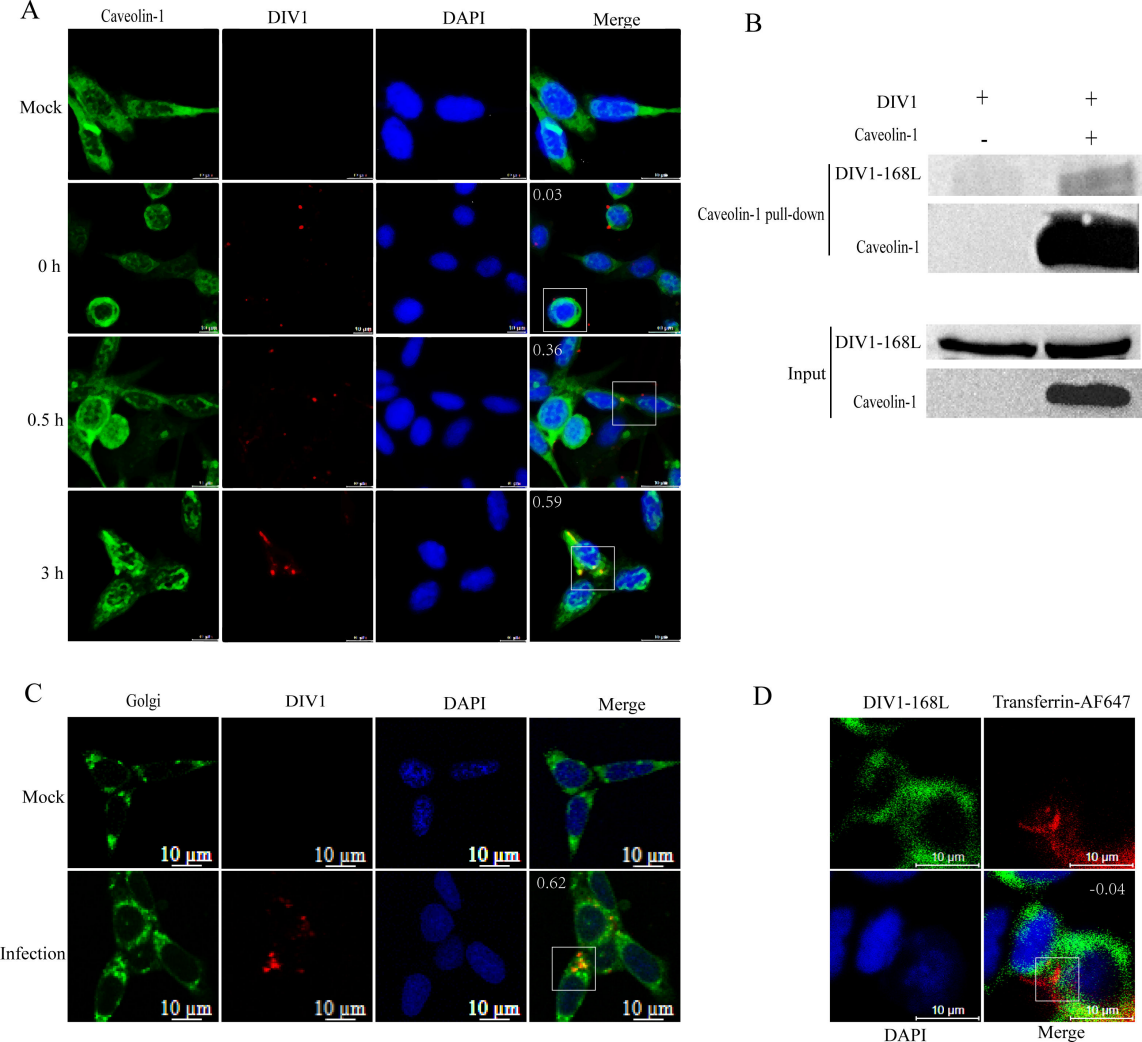
Confocal microscopy analysis of DIV1 internalization into HPT cells. (**A**) Time-course analysis of DIV1 internalization into HPT cells and its co-localization with caveolin-1. HPT cells were pre-chilled and incubated with Cy5-labeled DIV1 at 4°C for 1 h to allow binding. After removing the unbound virus, cells were shifted to 27°C to initiate internalization, then fixed with methanol at 0, 0.5, and 3 h post-internalization. IFA was performed using an anti-caveolin-1 antibody, followed by a FITC-conjugated secondary antibody. Uninfected cells served as a negative control. Confocal imaging was conducted, and the correlation between red fluorescence (DIV1) and green fluorescence (caveolin-1) was analyzed using ImageJ software. Pearson correlation coefficient (PCC) values are shown in the upper-left corner of the merged images. (**B**) Pull-down assays to verify the interaction between DIV1 and caveolin-1. Pull-down assays were performed with two groups: one using caveolin-1 as the bait protein, and the other without caveolin-1, with purified DIV1 included in both reactions. Eluted proteins from the caveolin-1 pull-down fraction and input samples were analyzed by WB. Blots were probed with antibodies against DIV1-168L or caveolin-1. (**C**) Localization of DIV1 in the Golgi apparatus. HPT cells were pre-chilled and co-incubated with DIV1 and NBD-labeled C6-ceramide (Golgi marker) at 4°C for 1 h to allow binding. Unbound substances were removed, and cells were shifted to 27°C for 4 h (to allow viral trafficking to the Golgi apparatus). After fixation with methanol and nuclear staining with DAPI, confocal imaging was performed. The correlation between red fluorescence (DIV1)-green fluorescence (Golgi) was analyzed using ImageJ software. PCC values are shown in the upper left corner of merged images. Scale bar: 10 μm. (**D**) Localization analysis of DIV1 and transferrin-AF647 during internalization. HPT cells were pre-chilled and co-incubated with DIV1 and transferrin-AF647 at 4°C for 1 h to allow binding. Unbound substances were removed, and cells were shifted to 27°C for 30 min to induce endocytosis. After fixation with methanol, IFA was performed to label DIV1 with an anti-DIV1-168L antibody, followed by a FITC-conjugated goat anti-rabbit antibody. Nuclei were counterstained with DAPI. Confocal images were acquired, and the correlation between red fluorescence (transferrin-AF647) and green fluorescence (DIV1) was analyzed via ImageJ software. PCC values are shown in the upper right corner of merged images. Scale bar: 10 μm.

To further confirm that DIV1 enters cells via CvME, we tracked the temporal dynamics of viral internalization using fluorescence microscopy. DIV1 was labeled with Cy5 (red fluorescence) to visualize viral particles, while caveolin-1 was immunolabeled with a rabbit polyclonal antibody, generated by immunizing rabbits with full-length caveolin-1 as the antigen and validated for specificity via WB (Fig. S1), to mark caveolar structures. Temporal analysis revealed that at 0 h, although viral particles bound to the cell surface, the Pearson correlation coefficient (PCC) (0.03) indicated no co-localization with caveolae (Fig. 7A), confirming that endocytosis had not yet initiated. At 0.5 h, the co-localization signal between Cy5-labeled DIV1 and caveolin-1 gradually intensified (PCC = 0.36), with significant overlap detected, verifying viral internalization through caveolae. By 3 h, most viral particles had entered cells and translocated to the perinuclear region, accompanied by a further increase in PCC (0.59) (Fig. 7A). Consistently, a pull-down assay provided biochemical evidence for the physical interaction between DIV1 and caveolin-1 (Fig. 7B). In the experimental group (with caveolin-1), DIV1-168L was clearly detected in the caveolin-1 pull-down fraction, whereas no 168L signal was observed in the negative control group (without caveolin-1). Input blots confirmed that DIV1 was equally loaded in both groups, and caveolin-1 was only present in the experimental group, ruling out non-specific binding artifacts. Together, the co-localization dynamics and direct protein-protein interaction demonstrate that DIV1 relies on caveolin-1-mediated CvME for internalization into host cells.

To further trace the post-internalization trafficking of DIV1, we analyzed its co-localization with the Golgi apparatus (labeled by NBD-C6-ceramide). In the mock group (uninfected cells), only the Golgi signal (green) was observed, with no DIV1 (red) detected (Fig. 7C). In DIV1-infected cells (incubated at 27°C for 4 h), the merged image showed significant overlapping signals (yellow) between DIV1 and the Golgi, supported by a high PCC (0.62) (Fig. 7C). This confirms that internalized DIV1 particles are transported to and co-localize with the Golgi apparatus.

To verify the specificity of the CvME pathway, we further analyzed the localization of DIV1 and transferrin-AF647. If DIV1 utilizes CvME, it should not co-localize with transferrin. After co-incubation and induction of internalization, a low PCC (−0.04) was observed between DIV1 and transferrin, with no obvious overlapping signals (Fig. 7D), confirming that the two use distinct internalization mechanisms.

Collectively, these results demonstrate that DIV1 enters HPT cells via CvME. It co-localizes with caveolin-1 but not transferrin and subsequently traffics to the Golgi apparatus, completing the early stage of viral internalization and intracellular transport.

## DISCUSSION

To explore DIV1’s entry into hematopoietic *C. quadricarinatus* cells, we investigated the roles of two major endocytic pathways, CME and CvME, using pathway-specific inhibitors. CME is widely utilized by viruses (e.g., African swine fever virus, human immunodeficiency virus type 1, and hepatitis B virus) for cellular entry (37–39). However, when we tested two validated CME inhibitors (pitstop 2, chlorpromazine), neither reduced DIV1 infection. This result rules out CME as a functional entry route for DIV1, distinguishing it from many well-characterized viruses and prompting us to investigate the alternative CvME pathway. We then targeted CvME by disrupting cholesterol-rich membrane microdomains (critical for caveola formation) with MβCD or nystatin: both inhibitors suppressed DIV1 internalization, and this effect was fully reversed by exogenous cholesterol replenishment. This cholesterol-dependent rescue not only confirms that DIV1 entry requires caveolae/lipid rafts but also aligns with the hallmark of CvME. Furthermore, CvME inhibitors (PMA, genistein, and wortmannin) markedly inhibited DIV1 infection, providing additional evidence that DIV1 hijacks CvME for entry. This conclusion was also validated by co-localization and pull-down assays. Specifically, the co-localization assay demonstrated that DIV1 co-localizes with caveolin-1, while the pull-down assay confirmed a direct physical interaction between DIV1 and caveolin-1 *in vitro*. This caveolin-1-dependent entry mechanism aligns with the conserved role of caveolins in iridovirid-host interactions. For example, TFV, ISKNV, and LCDV also rely on caveolin-1 to anchor to caveolae and initiate internalization (11–13), highlighting a shared adaptive strategy among iridovirids to exploit the structural stability of caveolae for efficient cell invasion. Importantly, caveolin-1 is not merely a passive “scaffold” for DIV1, but likely an active mediator of entry: its ability to interact with DIV1 may facilitate DIV1’s clustering into caveolar domains and subsequent recruitment of downstream CvME effectors. In addition, DIV1’s entry strategy also differs markedly from that of white spot syndrome virus (WSSV), another prevalent decapod virus. WSSV employs multiple endocytic pathways (CME, macropinocytosis, and CvME) to enter HPT cells of *C. quadricarinatus* (40).

Upon binding to cell-surface receptors, a tyrosine kinase signaling cascade recruits dynamin to internalization sites, coinciding with local actin cytoskeleton remodeling that enables dynamin’s association with caveolin-1 to facilitate co-endocytosis (41, 42). As the virus is enveloped by caveolae, dynamin-2 forms a GTP-dependent constrictive ring at the caveolar neck. GTP hydrolysis generates mechanical force to sever the plasma membrane, releasing free caveolar vesicles (35, 43). The role of dynamin in CvME has been well validated. Prior studies show that microinjection of anti-dynamin antibodies, expression of the dominant-negative dynamin K44A mutant, or dynasore-mediated inhibition of dynamin GTPase activity all block caveolae/raft-mediated internalization across various cellular models (36, 41, 44–46). Our observation that dynamin is required for DIV1 entry into HPT cells not only confirms that DIV1 hijacks canonical CvME machinery but also reinforces dynamin as a conserved factor in viral endocytosis, highlighting its potential as a broad-spectrum antiviral target.

Understanding viral trafficking routes is critical for unraveling fundamental viral-host interaction mechanisms and holds significant translational implications. Such insights can clarify viral pathogenesis (e.g., cellular invasion, immune evasion, and replication) and guide the development of antiviral drugs that disrupt these pathogenic pathways. CvME is a specialized cellular process with distinct intracellular trafficking routes. Ligands internalized via caveolae exhibit “fusion plasticity,” potentially merging with either CME-derived endosomes or caveosomes (2, 47). Caveosomes, caveolin-1-positive, pH-neutral organelles enriched in cholesterol and glycosphingolipids, form microenvironments distinct from canonical endosomal compartments. Their function (including cargo delivery and biological properties) is determined by the cargo itself. In non-infected cells, caveosomes primarily act as intermediate depots, facilitating the transport of sphingolipids and glycosylphosphatidylinositol-linked proteins from the plasma membrane to the Golgi apparatus. Cargo-specific trafficking divergence is well documented. For example, cholera toxin first enters caveosomes before trafficking to the Golgi complex and then the endoplasmic reticulum (ER), whereas autocrine motility factor bypasses caveosomes and is directly delivered to the ER (48). For viral cargo, caveosomes mediate further pathway specialization: viruses within caveosomes may be transported along microtubules to either the ER (e.g., SV40) or the Golgi apparatus (e.g., TFV) (2, 11). This divergence arises from ligand-specific traits, such as unique sorting signals and interactions with receptors or adaptor proteins on caveolar vesicles. In the Golgi-targeting pathway, the organelle’s low-pH environment triggers virion uncoating, enabling the release of viral DNA. Our study extends this understanding to DIV1. Via fluorescence co-localization assays, we confirmed that post-internalization, DIV1 exhibits significant signal overlap with the Golgi apparatus. This direct visual evidence validates that DIV1 traffics specifically to the Golgi after transit through caveosome, aligning with Golgi-targeting viruses like TFV (11), and differing from ER-targeted SV40 (2). Similar to other Golgi-targeting viruses, the Golgi’s mildly acidic pH is critical for DIV1 uncoating. Our data show that perturbing Golgi pH suppresses DIV1 infection, directly linking Golgi-mediated trafficking to the critical steps of viral DNA release and replication. This observation directly connects Golgi-mediated trafficking to the critical steps of viral DNA release and replication. Unresolved questions remain, including the identity of DIV1’s Golgi-targeting sorting signals and how DIV1 modulates cellular trafficking machinery. Answering these questions could advance our understanding of both viral pathogenesis and crustacean caveosome-Golgi dynamics. For example, revealing whether DIV1 hijacks caveosomal sphingolipid transport pathways or uses viral proteins to interact with Golgi adaptors. Such insights would inform the development of targeted therapies for DIV1 control in decapod aquaculture.

As a crucial cytoskeletal component, microtubules form a dynamic network that acts as “tracks” for motor proteins (e.g., kinesin and dynein) to transport vesicles and organelles, supporting intracellular trafficking and cell shape maintenance. This transport function is not only fundamental to general intracellular order but also specifically critical for caveolar vesicle movement (49). Our experiments further validated and expanded this mechanism. First, after internalization, the intracellular transport of caveolar vesicles depends entirely on an intact microtubule network. Treatment with nocodazole completely blocked this transport process. Second, we found that the role of microtubules is mainly restricted to the early viral entry stage (0–1 hpi) and has no direct impact on subsequent processes like viral uncoating and replication. Specifically, DIV1 relies on microtubules to be transported to the Golgi apparatus via caveolar vesicles post-internalization. Disrupting microtubules during the 0–1 hpi window severs this “transport track,” blocking the DIV1-carrying caveolar vesicles trafficking process, and thereby depriving DIV1 of the cellular environment required for subsequent replication. In contrast, once virus-carrying vesicles reach the Golgi, microtubules become dispensable for subsequent steps, explaining why nocodazole intervention at 2–4 hpi fails to inhibit infection.

**Fig 8:**
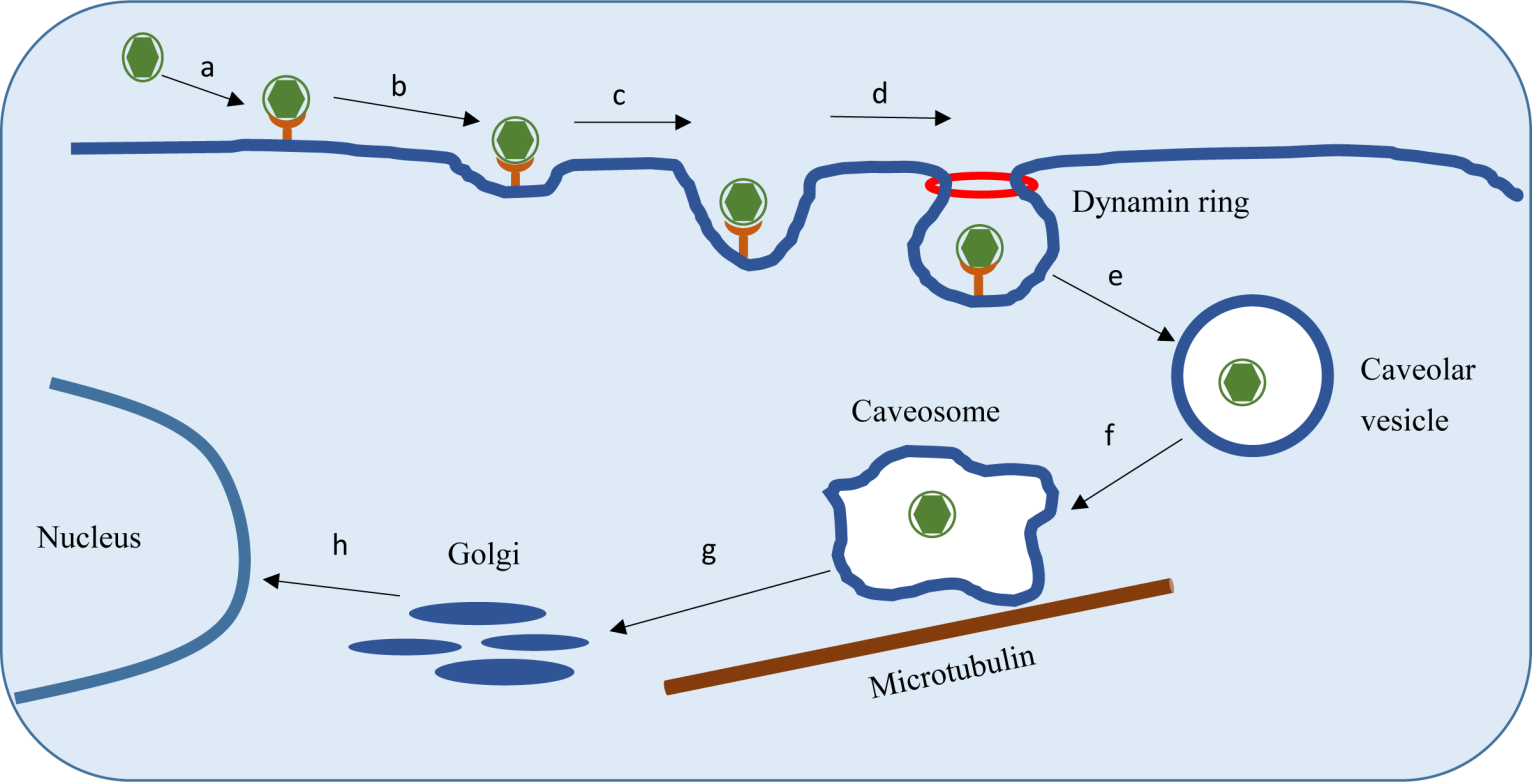
Schematic model of the entry route of DIV1 into HPT cells. (a) DIV1 virions bind to the receptor on the cell surface. (b and c) The binding signal between DIV1 and the cell receptor induces the formation of caveolae at the binding site. (d) Dynamin is recruited to the caveolae-mediated internalization site, whereas it mediates the budding of caveolar invaginations from the plasma membrane. (e) Caveolar invaginations containing DIV1 virions are pinched off from the plasma membrane, forming free caveolar transport vesicles. (f) The free caveolar transport vesicles mature and are converted into caveosomes. (g) Caveosomes containing DIV1 are transported along the microtubule network to the Golgi apparatus. (h) Upon reaching the Golgi apparatus, DIV1 gradually undergoes uncoating and capsid disassembly, followed by the release of its genome into the cell nucleus to complete the entry process.

Overall, these results consistently demonstrate that DIV1 enters hematopoietic *C. quadricarinatus* cells through a CvME pathway dependent on cholesterol, caveolin-1, and dynamin. Post-internalization, DIV1 is transported to caveosomes and subsequently trafficked to the Golgi apparatus via a microtubule-dependent system (Fig. 8). The mildly acidic pH environment of the Golgi is crucial for DIV1 particle uncoating, and any perturbation of Golgi pH can disrupt this process, inhibiting viral infection.

It is important to note that the entry mechanism characterized in this study is specific to the HPT cells of *C. quadricarinatus*. Viral entry pathways are frequently cell type-dependent, a phenomenon well documented in the family *Iridoviridae* and other viral taxa. For example, different iridovirids (e.g., TFV, SGIV) utilize distinct entry routes when infecting diverse host cell types, and even a single virus (e.g., porcine deltacoronavirus) can switch between endocytic pathways (CME, CvME) depending on the target cell line (7, 11, 50, 51). Extending this context to DIV1, it is likely that DIV1 will adopt alternative entry mechanisms when infecting cells derived from other tissues of *C. quadricarinatus*. This aligns with the broader observation that viral entry plasticity allows pathogens to adapt to the unique cellular environments of different host tissues, a strategy that likely supports efficient infection and dissemination *in vivo*. Thus, our current findings on CvME-dependent entry should not be generalized to all *C. quadricarinatus* cell types. Future studies targeting non-hematopoietic cells (e.g., epithelial, muscle cells) of this crayfish or other host species will be critical to fully elucidate the tissue- and host-specific entry strategies of DIV1.

## MATERIALS AND METHODS

### Cell isolation and culture

HPT cells were freshly isolated from the healthy adult *C. quadricarinatus* following a previously established protocol (20). Briefly, hematopoietic tissues were enzymatically digested using type I and type IV collagenases (Sangon, CHN). The digested cell suspension was filtered through a cell strainer (Sangon, CHN) to remove undigested tissue fragments and cell clumps, yielding a single-cell suspension. Isolated cells were cultured in Leibovitz’s L-15 medium (Gibco, USA) supplemented with 10% fetal bovine serum (Gibco, USA) at 27°C.

### Virus isolation, propagation, and purification

The DIV1 strain (GenBank accession no. MF197913) used in this study was initially isolated from diseased *C. quadricarinatus* (17). For propagation, healthy *C. quadricarinatus* were intra-abdominally injected with homogenates of DIV1-infected tissue. Hemolymph was collected from moribund crayfish to prepare virus stocks. For purification, hemolymph containing DIV1 was centrifuged at 3,000 × *g* for 15 min, and the supernatant was filtered through a 0.45 μm filter (Millipore, USA). A CsCl density gradient was prepared by layering 8 mL of 10 mM Tris-HCl (pH 8.0) with CsCl (density 1.4 g/mL) at the bottom of a centrifuge tube, followed by 8 mL of the same buffer with CsCl (density 1.2 g/mL) on top. The virus supernatant was carefully overlaid onto the CsCl gradient and ultracentrifuged at 71, 000 × *g* for 2 h at 4°C. The DIV1 band was collected, mixed with an equal volume of PBS (pH 7.4), and ultrafiltered three times using a 50 kD ultrafilter (Millipore, USA). The purified virus was aliquoted and stored at −80°C.

### Kinetics of DIV1 infection and cellular entry

To characterize the kinetics of DIV1 infection, HPT cells were infected with DIV1 at a multiplicity of infection (MOI) of 10 at 27°C. At 0, 1, 2, 3, 4, 5, 6, 7, and 8 hpi, non-internalized viruses were inactivated and removed by washing cells once with citrate buffer (40 mM sodium citrate, 10 mM KCl, 135 mM NaCl, pH 3.0) followed by three washes with PBS (52). Fresh medium was then added, and cells were cultured for an additional 72 h.

To analyze the kinetics of DIV1 entry, HPT cells were pre-chilled at 4°C and incubated with DIV1 (MOI = 10) at 4°C for 30 min to allow viral binding to the cell surface. The unbound virus was removed by three washes with pre-chilled PBS. Fresh medium was added, and cells were immediately transferred to 27°C to trigger internalization of bound virus. At 0, 0.5, 1, 1.5, 2, 2.5, 3, 3.5, and 4 h post-endocytosis initiation, non-internalized viruses were removed via citrate buffer and PBS, and cells were cultured for 72 h.

Viral copy numbers in the culture medium were quantified by qPCR. For data normalization and comparative analysis, two distinct baseline time points were set. In the infection kinetics assay, the DIV1 copy rate at 8 hpi was set as the reference (100%), while in entry the kinetics assay, the DIV1 copy rate at 4 h post-endocytosis was set as the reference (100%). Relative viral abundance at other time points was calculated as a percentage of this reference.

### Fluorescence labeling of DIV1

Purified DIV1 was diluted in 0.1 M sodium bicarbonate buffer (pH 8.3), and a 5 mM Cy5-NHS ester stock solution (dissolved in DMSO, Solarbio, CHN) was added dropwise to the virus suspension at a molar ratio of 10: 1 (Cy5-NHS ester to virus particles) with continuous gentle stirring. The reaction mixture was incubated at room temperature for 2 h with gentle vortexing. Unbound Cy5 was removed by dialysis against PBS (pH 7.4) at 4°C overnight. Labeled DIV1 was assessed for fluorescence intensity and purity and stored at −80°C in the dark.

### Preparation of caveolin-1 antibody

For *C. quadricarinatus,* its caveolin-1 coding sequence (GenBank accession no. NC_091351.1) was inserted into the vector pET-28a (+) via homologous recombination, using the primers: forward 5′-AGCAAATGGGTCGCGGATCCATG

GGTGGTGGTACCCCTAG-3′ and reverse 5′-TGGTGGTGGTGGTGCTCGAGTTA

AACGTTAAAATAATCGCTACTTTG-3′. The recombinant vector encoded a caveolin-1 carrying an N-terminal His tag with a predicted molecular weight of 20.4 kDa. Subsequently, the recombinant caveolin-1 protein was expressed in *Escherichia coli* BL21 (DE3) cells and purified via Ni-NTA affinity chromatography (Beyotime, CHN). Six New Zealand white rabbits (approximately 2.5 kg each) were acclimated for 2 weeks before immunization. For the primary immunization, 0.5 mg of antigen was emulsified with Freund’s complete adjuvant (Sigma, USA) at a 1: 1 (vol/vol) ratio and subcutaneously injected at 8 dorsal sites. Three boosters were administered at 2-week intervals, each using 0.25 mg of antigen emulsified with Freund’s incomplete adjuvant (Sigma, USA). Rabbit serum was collected 7 days after the third booster. Specific anti-caveolin-1 antibodies were purified using a CNBr-activated Sepharose 4B column (GE Healthcare, USA) coupled with caveolin-1. Eluted antibodies were neutralized, dialyzed against PBS (pH 7.4), and concentrated to 11.2 mg/mL, and the specificity was validated by WB using total protein extracts from HPT cells.

### Inhibitor assays

Chemical inhibitors (Sigma-Aldrich, USA) targeting endocytic pathways were prepared as follows: pitstop 2, methyl-β-cyclodextrin (MβCD), chlorpromazine, nystatin, dynasore, phorbol 12-myristate 13-acetate (PMA), genistein, bafilomycin A1, nocodazole, and wortmannin were dissolved in DMSO. NH_4_Cl was dissolved in sterile double-distilled water (ddH_2_O). Stock solutions were stored at −80°C.

To assess the potential cytotoxicity of the inhibitors, the CCK-8 assay was performed. Cells were seeded in 96-well plates at a density of 4 × 10^4^ cells/well and incubated overnight. Cells were treated with each inhibitor at the following concentrations: chlorpromazine (5, 10, 15, 20, and 30 μM), pitstop 2 (5, 10, 15, 20, and 30 μM), MβCD (0.5, 1, 2, 2.5, and 3 mM), nystatin (25, 50, 75, 100, and 125 μg/mL), PMA (1, 2, 5, 10, and 15 μM), genistein (10, 25, 50, 75, and 100 μM), wortmannin (10, 25, 50, 100, and 125 μM), bafilomycin A1 (50, 100, 150, 200, and 300 nM), NH_4_Cl (10, 25, 50, 75, and 100 mM), dynasore (10, 25, 50, 80, and 100 μM), and nocodazole (1, 2, 5, 10, and 15 μM) for 5 h at 27°C. Control cells included both solvent-treated groups (receiving an equal volume of DMSO or ddH_2_O) and an untreated blank control group. After treatment, 10 μL of CCK-8 reagent (Beyotime, CHN) was added to each well (including blank wells containing only cell culture medium and CCK-8 reagent, without any cells), and the plates were incubated at 37°C for 2 h. Absorbance at 450 nm was measured using a microplate reader (Tecan, CH). Cell viability was calculated as follows: Cell Viability (%) = (absorbance of treated cells − absorbance of blank wells)/(absorbance of untreated control cells-absorbance of blank wells) × 100. Data are representative of three independent experiments performed in triplicate.

To validate the efficacy of inhibitors in suppressing endocytosis, HPT cells were treated with pitstop 2 (20 μM), chlorpromazine (20 μM), NH_4_Cl (50 mM), and bafilomycin A1 (200 nM) at 27°C for 1 h. Solvent-treated cells alongside untreated cells served as controls. Subsequently, Alexa Fluor 647-conjugated transferrin (transferrin-AF647, Invitrogen, USA) was added to a final concentration of 10 μg/mL, and cells were incubated for another 1 h. Cells were washed with citrate buffer and PBS, stained with DAPI, and imaged using a confocal microscope (Leica, GER). For inhibiting CvME, cells were treated separately with MβCD (5 mM), nystatin (100 μg/mL), PMA (10 μM), genistein (50 μM), and wortmannin (100 μM) 27 ℃ for 1 h. Solvent-treated cells served as controls. Following this, Alexa Fluor 647-conjugated CTB (CTB-AF647, Invitrogen, USA) was added to a final concentration of 20 μg/mL, and cells were incubated for another 1 h. Cells were processed (washing, DAPI staining) and imaged via confocal microscope as described above. To verify the effect of the inhibitor on viral internalization, cells were pretreated with inhibitors at 27°C for 1 h. DIV1 was then added at an MOI of 10, followed by incubation at 27°C for 4 h. Positive control cells were infected with the same dose of DIV1 but only supplemented with the solvent. Negative control cells were not infected with DIV1. Non-internalized viruses were removed via citrate buffer and PBS, and cells were cultured in fresh medium for 72 h. Viral copy number in culture medium was quantified by qPCR, and intracellular DIV1-168L protein expression was detected by WB.

### Cholesterol replenishment assay

Cells were co-treated with MβCD (2 mM) and exogenous cholesterol (0, 10, 25, 50, and 75 μg/mL, Sigma-Aldrich, USA) at 27 ℃ for 1 h, followed by infecting with DIV1 (MOI = 10) for 4 h. Subsequently, the cells were treated identically as described in the above section labeled “Inhibitor assay.”

### Quantitative polymerase chain reaction

Viral DNA was extracted from culture medium using the UNlQ-10 column virus genomic DNA isolation kit (Sangon Biotech, CHN). qPCR was performed on a CFX96 Real-time System (BIO-RAD, USA) with primers targeting the DIV1 *037L* region, which is predicted to encode a D5-like primase: forward 5′-AGGAGAGGGAAATAACGGGAAAAC-3′ and reverse 5′-CGTCAGCATTTGGTTCATCCATG-3′. The 10 μL reaction system contained 5 μL 2×SYBR Green PCR Master Mix (BIO-RAD, USA), 0.5 μM of each primer, 1 μL DNA template, and ddH_2_O to reach the final volume. Cycling conditions were as follows: initial denaturation at 95°C for 5 min, followed by 40 cycles of denaturation at 95°C for 15 s and annealing/extension at 60°C for 30 s, with a melting curve analysis (60°C–95°C at 0.5°C/s). Viral copy number was calculated using a standard curve generated with a serial dilution of a pMD19-T vector containing the DIV1 *037L* gene, with triplicate reactions per sample.

### Western blotting

Adherent cells were lysed in RIPA buffer (Beyotime, CHN) supplemented with protease/phosphatase inhibitors (Beyotime, CHN) on ice for 15 min and centrifuged at 14,000 × *g* for 10 min at 4°C. Supernatants were denatured in SDS loading buffer at 95°C for 5 min, separated by SDS-PAGE, and transferred to nitrocellulose membranes. Membranes were blocked with 5% non-fat milk in TBST (50 mM Tris, 150 mM NaCl, 0.05% Tween 20) for 1 h, then incubated overnight at 4°C with DIV1-168L antibody (1:1,000 dilution in 5% milk-TBST). After three washes with TBST, membranes were incubated with horseradish peroxidase (HRP)-conjugated goat anti-rabbit secondary antibody (1:5,000 dilution in 5% milk-TBST, Promega, USA) at room temperature for 1 h. Signals were detected using a chemiluminescent HRP substrate (Coolaber, CHN) and imaged with a chemiluminescence imaging system (P&Q Science & Technology, CHN). Membranes were stripped and reprobed with an anti-β-actin antibody (1:1,000, TransGen Biotech, CHN) for normalization. Experiments were performed in replicate.

### Localization analysis of DIV1 with caveolin-1, transferrin-AF647, and the Golgi apparatus

For DIV1-caveolin-1 localization analysis, cells were pre-chilled and incubated with Cy5-labeled DIV1 at 4°C for 1 h. The unbound virus was removed by three PBS washes, and the cells were transferred to 27°C to initiate endocytosis. At 0, 0.5, and 3 h post-endocytosis, cells were fixed with pre-chilled methanol for 10 min, and IFA was performed using anti-caveolin-1 antibody. Co-localization of Cy5-DIV1 and caveolin-1 was analyzed via confocal microscopy (Leica, GER), with PCC calculated using ImageJ software. Uninfected cells served as negative controls.

For DIV1-Golgi localization analysis, pre-chilled cells were co-incubated with DIV1 and NBD-C6-ceramide (Beyotime, CHN) at 4°C for 1 h to allow binding. Unbound substances were removed by three PBS washes, and cells were transferred to 27°C for 4 h to promote DIV1 trafficking to the Golgi apparatus. Cells were fixed with methanol, stained with DAPI, and imaged via confocal microscope (Leica, GER). PCC was calculated using ImageJ software. Uninfected cells served as negative controls.

For DIV1-transferrin-AF647 localization analysis, pre-chilled cells were co-incubated with DIV1 and transferrin-AF647 at 4°C for 1 h to enable binding. Unbound substances were removed by three PBS washes, and cells were incubated at 27°C for 30 min to induce endocytosis. Cells were fixed with methanol, and IFA was performed using anti-DIV1-168L antibody. Localization of DIV1 and transferrin-AF647 was imaged via confocal microscope (Leica, GER), and PCC was calculated using ImageJ software.

### Immunofluorescence

After washing three times with PBS, the fixed cells were permeabilized with 0.5% Triton X-100 (dissolved in PBS) at room temperature for 15 min, followed by three additional washes with PBS. Next, the cells were blocked with 10% normal goat serum (in PBST) at room temperature for 1 h. After the blocking step, the cells were incubated overnight at 4°C with anti-caveolin-1 antibody or anti-DIV1-168L antibody (both diluted at a ratio of 1:500 in 1% normal goat serum). On the following day, the primary antibody solution was carefully aspirated, and the cells were washed three times with PBST. Subsequently, the cells were incubated at room temperature for 1 h with a fluorescein isothiocyanate (FITC)-conjugated goat anti-rabbit antibody (Bioss, China), which was diluted 1:500 in 1% normal goat serum. After another three washes with PBST, the cells were stained with DAPI (Bio Basic, CAN) to visualize cell nuclei. Finally, the samples were imaged using a confocal microscope (Leica, GER).

### Pull-down assay

Recombinant caveolin-1 was expressed and purified as described in the section of “Preparation of caveolin-1 antibody.” Protein A/G magnetic beads (Beyotime, CHN) were incubated with 5 μg/mL caveolin-1 antibody at room temperature for 1 h. After washing with TBS (50 mM Tris-HCl, 150 mM NaCl, pH 7.4) to remove unbound antibody, the beads were divided into two groups: one mixed with purified caveolin-1 (experimental group) and the other without caveolin-1 (negative control group), with purified DIV1 added to both groups. The mixtures were incubated at 4°C overnight with gentle rotation. Beads were washed three times with TBS, and bound proteins were eluted by boiling the beads in 1× SDS-loading buffer for 5 min. Eluted proteins and input samples were analyzed by WB using antibodies against DIV1-168L or caveolin-1.

## Data Availability

All data generated and analyzed in this study are included in this article and its supplementary material. Other raw data supporting the conclusions are available from the corresponding author upon reasonable request.

## SUPPLEMENTAL MATERIAL

Figure S1 jvi.01681-25-s0001.docx Western blot analysis of caveolin-1 antibody specificity in Cherax quadricarinatus hematopoietic tissue (HPT) cells.

ASM does not own the copyrights to Supplemental Material that may be linked to, or accessed through, an article. The authors have granted ASM a non-exclusive, world-wide license to publish the Supplemental Material files. Please contact the corresponding author directly for reuse.
