# Supplementary material for: Decapod iridescent virus 1 (DIV1) enters hematopoietic Cherax quadricarinatus cells via caveola-mediated endocytosis in a pH-dependent manner
Source: J Virol. 2026 Mar 30;100(4):e01681-25. doi: 10.1128/jvi.01681-25 (PMC13098244; doi:10.1128/jvi.01681-25)
Supplement: Figure S1 — Western blot analysis of caveolin-1 antibody specificity in Cherax quadricarinatus hematopoietic tissue (HPT) cells. [file jvi.01681-25-s0001.docx]

A

B

**
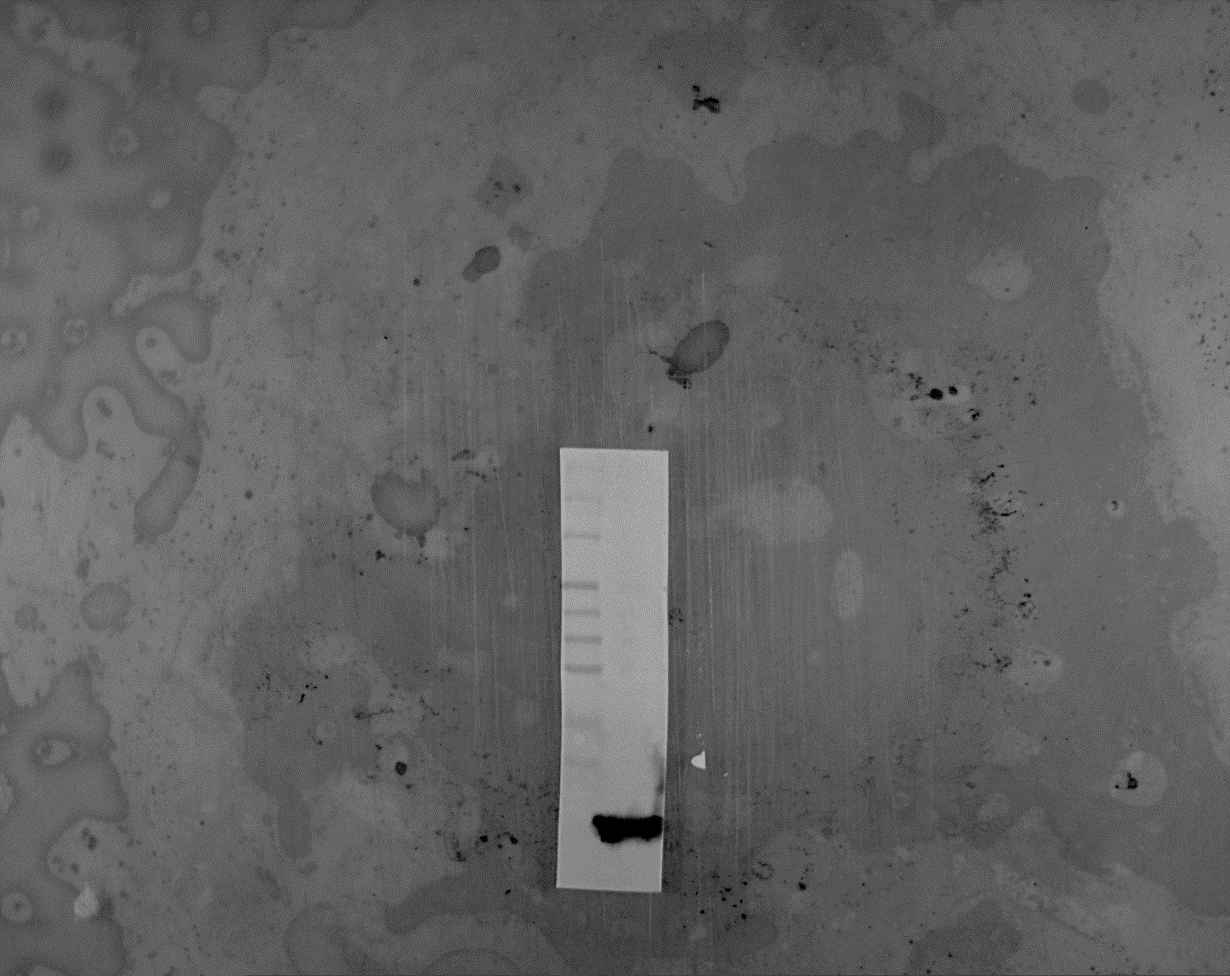

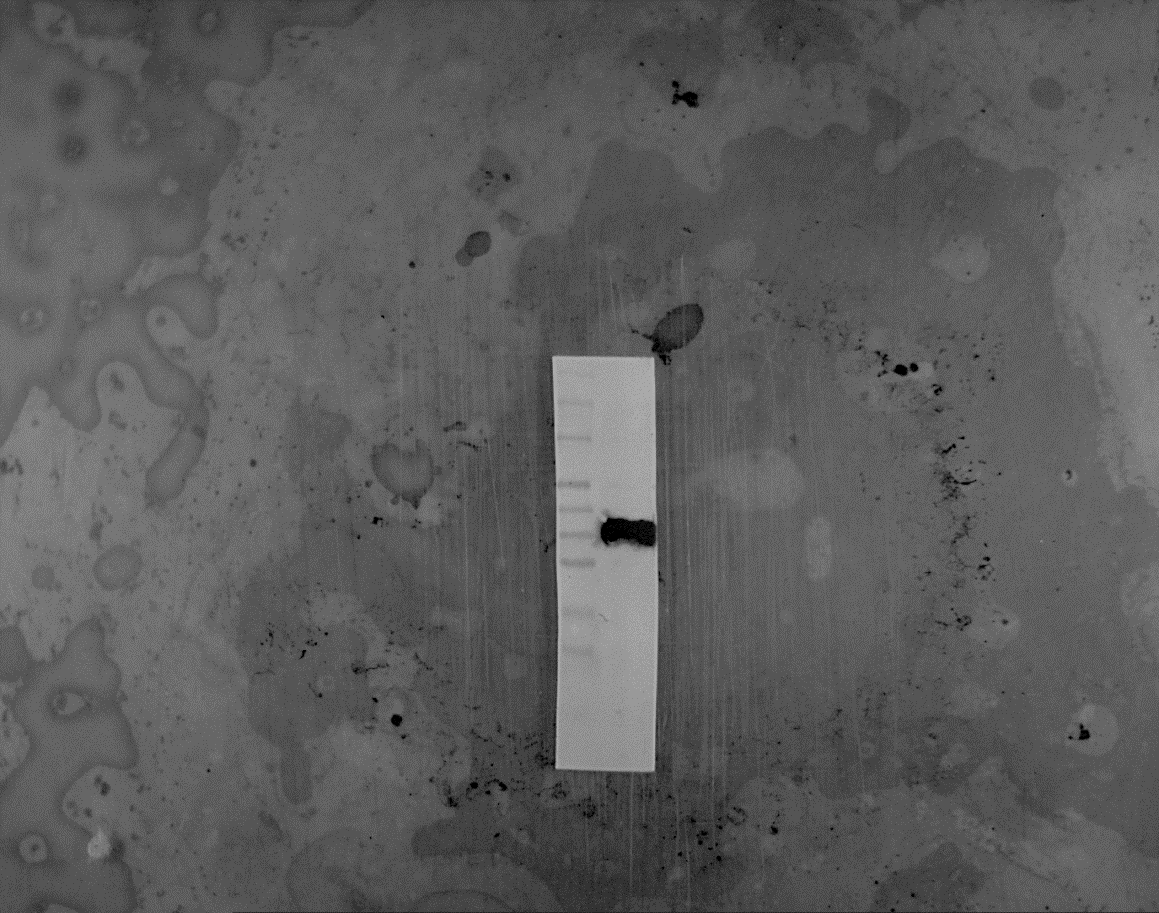
**

HPT

250-

kDa

100-

150-

70-

50-

40-

35-

20-

25-

40-

50-

15-

35-

HPT

kDa

**
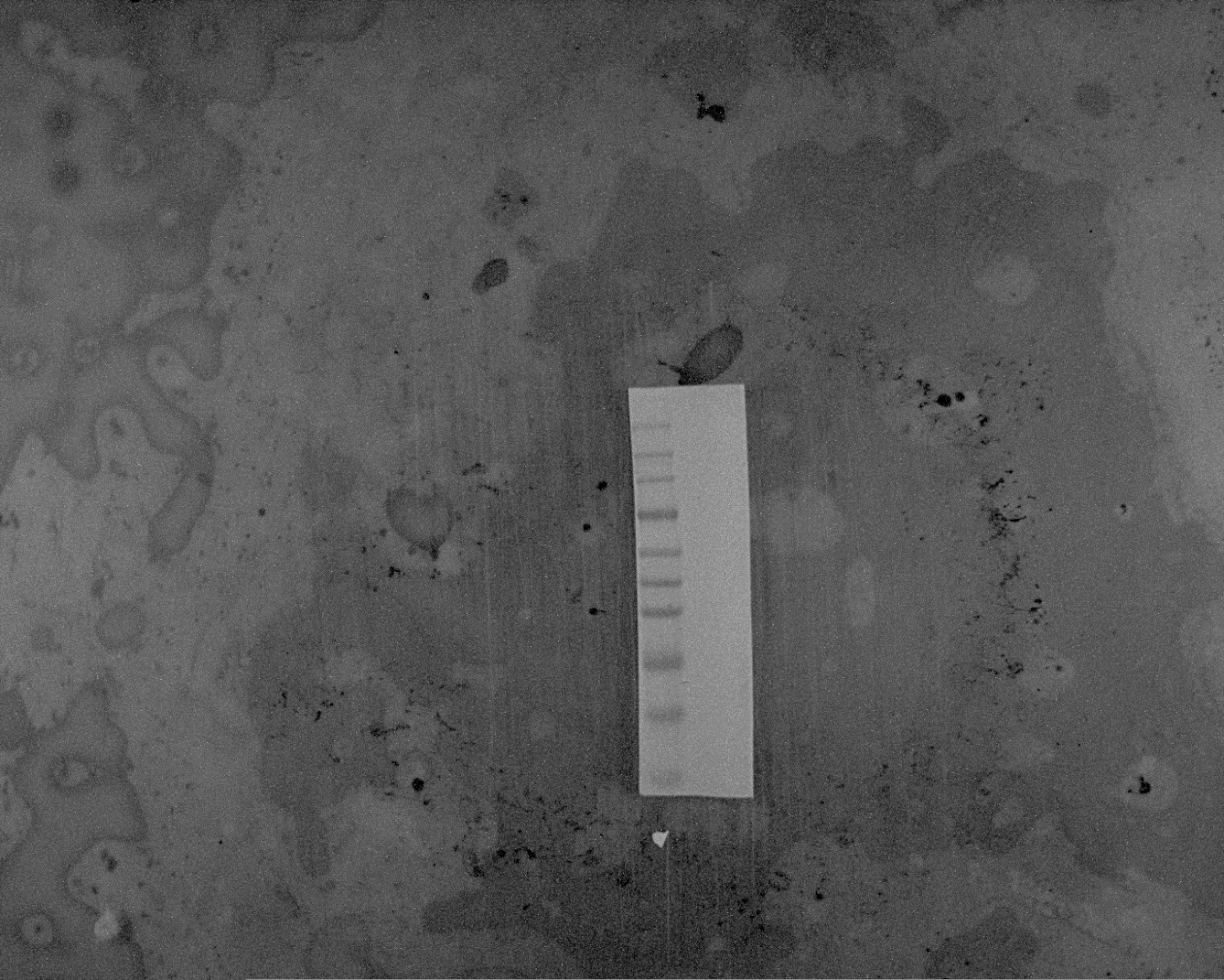
**

150-

250-

70-

100-

40-

50-

25-

35-

20-

15-

**
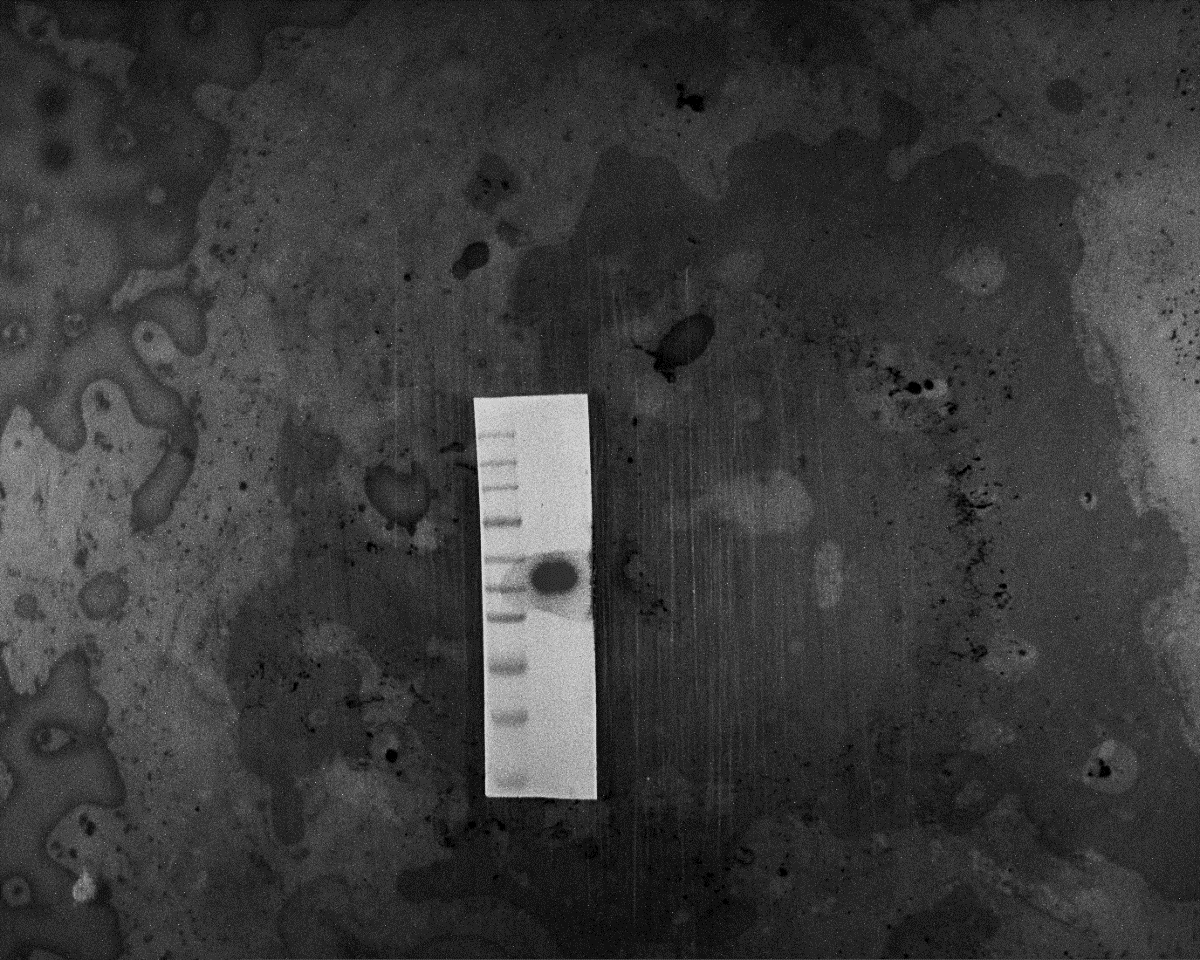
**

35-

50-

40-

**Supplementary Figure 1. Western blot analysis of caveolin-1 antibody** **specificity in *Cherax quadricarinatus* hematopoietic tissue (HPT) cells.** (A) Total proteins extracted from *C. quadricarinatus* HPT cells were subjected to Western blot analysis using a caveolin-1 antibody. (B) Negative control: The experiment was conducted under the same conditions as (A), except that the caveolin-1 antibody was substituted with pre-immune rabbit serum to assess non-specific binding.
